# A Cross-Layer Optimized Opportunistic Routing Scheme for Loss-and-Delay Sensitive WSNs

**DOI:** 10.3390/s18051422

**Published:** 2018-05-03

**Authors:** Xin Xu, Minjiao Yuan, Xiao Liu, Anfeng Liu, Neal N. Xiong, Zhiping Cai, Tian Wang

**Affiliations:** 1 School of Information Science and Engineering, Central South University, Changsha 410083, China; XinXuTec@csu.edu.cn (X.X.); yuanminjiao@csu.edu.cn (M.Y.); afengliu@mail.csu.edu.cn (A.L.); 2 The State Key Laboratory of Industrial Control Technology, Zhejiang University, Hangzhou 310027, China; 3 Department of Mathematics and Computer Science, Northeastern State University, OK 74464, USA, xiongnaixue@gmail.com; 4 Department of Network Engineering, School of Computer, National University of Defense Technology, Changsha 410073, China; zpcai@nudt.edu.cn; 5 School of Computer Science, National Huaqiao University, Quanzhou 362000 China; wangtian@hqu.edu.cn

**Keywords:** wireless sensor networks, opportunistic routing, transmission power, delay, reliability

## Abstract

In wireless sensor networks (WSNs), communication links are typically error-prone and unreliable, so providing reliable and timely data routing for loss- and delay-sensitive applications in WSNs it is a challenge issue. Additionally, with specific thresholds in practical applications, the loss and delay sensitivity implies requirements for high reliability and low delay. Opportunistic Routing (OR) has been well studied in WSNs to improve reliability for error-prone and unreliable wireless communication links where the transmission power is assumed to be identical in the whole network. In this paper, a Cross-layer Optimized Opportunistic Routing (COOR) scheme is proposed to improve the communication link reliability and reduce delay for loss-and-delay sensitive WSNs. The main contribution of the COOR scheme is making full use of the remaining energy in networks to increase the transmission power of most nodes, which will provide a higher communication reliability or further transmission distance. Two optimization strategies referred to as COOR(R) and COOR(P) of the COOR scheme are proposed to improve network performance. In the case of increasing the transmission power, the COOR(R) strategy chooses a node that has a higher communication reliability with same distance in comparison to the traditional opportunistic routing when selecting the next hop candidate node. Since the reliability of data transmission is improved, the delay of the data reaching the sink is reduced by shortening the time of communication between candidate nodes. On the other hand, the COOR(P) strategy prefers a node that has the same communication reliability with longer distance. As a result, network performance can be improved for the following reasons: (a) the delay is reduced as fewer hops are needed while the packet reaches the sink in longer transmission distance circumstances; (b) the reliability can be improved since it is the product of the reliability of every hop of the routing path, and the count is reduced while the reliability of each hop is the same as the traditional method. After analyzing the energy consumption of the network in detail, the value of optimized transmission power in different areas is given. On the basis of a large number of experimental and theoretical analyses, the results show that the COOR scheme will increase communication reliability by 36.62–87.77%, decrease delay by 21.09–52.48%, and balance the energy consumption of 86.97% of the nodes in the WSNs.

## 1. Introduction

Wireless sensor networks (WSNs) are composed of inexpensive microprocessors with wireless communication, computing, storage capabilities and the ability to perceive the surroundings [1,2,3,4]. They are emerging as promising platforms that enable a wide range of applications in numerous areas such as smart cities [5,6], traffic monitoring [7], factory automation control [8], public facilities monitoring [9,10], human health monitoring [11,12], weather monitoring, wildlife protection and military applications [13,14,15,16,17,18,19]. With the development of micro-processing technology, sensor nodes are not only becoming more and more powerful, but also have a smaller volume and cheaper price. Consequently, the prospects of sensor nodes are more and more promising [20,21,22], such as forming a new type of edge computing and edge network [5,23]. Besides, they become an important part of the Internet of Things (IoT) [2,12,18,24,25], which enables different Internet-connected objects to exchange data. Combined with cloud networks [4,15,26], Sensor-Cloud Networks (SCNs) can also be made up of those sensors. Furthermore, there is a growing interest in forming social networks [24,27,28] with mobile sensor devices like mobile phones, which can deeply change human life. Additionally, collecting data from monitored objects [29,30,31] is one of the important applications of wireless sensors, in which sensor nodes form a dynamic topological network by self-organization, and transmit sensed data to a control center (called sink) by multi-hop routing [32,33,34]. Then the sink performs the corresponding control action on the monitored or controlled objects so as to achieve some specific functions [35,36,37,38]. As well known, there are numerous applications requiring high transmission reliability for data routing [29,36]. The transmission reliability is particularly important for mission-critical applications such as remote patient monitoring, battlefield monitoring, monitoring of disaster-struck regions, home automation, and tracking of chemical and explosive agents. These applications are loss-and-delay sensitive and therefore reliable and timely delivery of information is critical [39,40,41]. In addition to loss-and-delay sensitivity, the energy efficiency is also of concern because wireless sensor nodes have limited battery capacity [42,43].

However, because of low power radios, noise, multipath interference and external interference [44,45], wireless communication links in WSNs are typically volatile, unreliable, and error-prone [37]. Unfortunately, bad links in a routing path may lead to unpredictable data packet loss. Even worse, the sink it is more likely to make wrong decisions without sufficient data. In addition, even if a retransmission policy is applied, it may cause increased end-to-end delay, which may further bring about system loss due to unresponsiveness to emergencies in a timely way and more energy consumption, thus reducing the network lifetime.

There are already a number of reliable, low delay and energy efficient routing strategies for WSNs [29,36,46]. The one-hop transmission reliability of data between two sensor nodes mainly refers to the probability that the receiver node successfully receives data after the sender node sends packets. Considering that in WSNs, the data generated by the source node is usually routed to the sink by multiple hops, therefore end-to-end reliability indicates the probability that the sink successfully receives the data packet after passing through multiple hops, hence end-to-end reliability is the product of all the one-hop transmission reliabilities on the routing path [47]. Accordingly, if one-hop transmission reliability is determined, end-to-end reliability will decrease with the growth of the number of routing hops. As a consequence, in order to maintain high reliability in a long route, it is necessary to maintain a high one-hop transmission reliability [29,47]. The reliability strategies that have been proposed are mainly as follows: (1) Data retransmission mechanism. The send-wait protocol [47] is the most representative method, whose main feature is confirming the transmission status of the packet by another special message called acknowledgement (ACK). (2) Multipath routing approach [48]. In this approach, a node transmits multiple copies of data and routes to destinations along different paths, so data routing fails only if all routes fail. Compared to a data retransmission mechanism, multipath routing provides an advantage of smaller delays. (3) Data encoding method [46]. The mechanism of this method is that the data is encoded in advance of being routed. With the help of redundant code, the receiver can deduce the missing data in incomplete received information, thus maintaining a certain reliability in the volatile WSNs communication links. However the main drawback is that encoding increases the amount of data, which means more energy consumption and reduced network life. (4) Opportunistic routing scheme [49]. In this way, instead of directly determining the next hop node, sender nodes determine a set of candidate nodes with higher-priority selected from the neighbor nodes, and then broadcast packets to these candidate nodes. This kind of routing method avoids the missing data caused by unexpected anomaly of nodes in the path, and improves the transmission reliability.

Similarly, delays can also be divided into one-hop delays and end-to-end delays [47]. The former indicates the period of time during the sender node begins sending data to the receiver until the receiver node receives that data. The latter is the time interval of transmission from source node to sink through multi-hop routing. Generally speaking, there are plenty of factors affecting delay, but the reliability of communication links plays a decisive role. The strategies adopted in reliability assurance often determine the delay. Thus, in many studies, delays are often studied in conjunction with reliability, but rarely studied separately.

The reliable and low delay guarantee methods mentioned above mainly improve the communication reliability from the perspective of the network layer. Making full use of the characteristics of wireless broadcast transmission, the sender only needs to send data once and high reliability is ensured through an opportunistic routing scheme. However, optimizing only the network layer will provide a very limited performance improvement. Actually, the network reliability and delay can be optimized not only from the network layer, but also from many other aspects. Moreover, comprehensive optimization of multiple layers can always get better results. Based on the above analysis, a Cross-layer optimized Opportunistic Routing (COOR) scheme is proposed in this paper. Different from previous research, the main innovations of our work are as follows:

(1) A Cross-layer optimized Opportunistic Routing (COOR) scheme is proposed to improve reliability of communication links and reduce delays for loss-and-delay sensitive WSNs. The main innovation of the COOR scheme is that rather than optimizing only the network layer, the performance of the network is improved by increasing the transmission power of the nodes with remaining energy and combining the routing method, so as to improve the performance of the network more effectively.

(2) Two kinds of cross layer optimization methods are given to improve network performance effectively in this paper. The first one selects the same candidate node set as the previous strategy for routing after increasing the transmission power of the nodes with remaining energy. With the improvement of transmission power, the reliability of the links is promoted. As a result, even if the routing scheme is the same as the previous one, the routing is still more stable. Moreover, the increase in reliability reduces the chance of queuing and validating between candidate nodes, thus reducing latency. Another approach is to select a new candidate nodes set with the same reliability as the previous strategy. In this way, the improvement of transmission power results in farther transmission distance, which means fewer hops are needed to reach the sink. Consequently, it satisfies the need of reducing delay and improving reliability.

(3) We provide an extensive theoretical analysis to confirm the strong effectiveness of our scheme. In our experimental comparison, for medium scale sensor networks, our scheme can improve the reliability by 36.62% and reduce delay by about 21.09%.

The rest of the paper is organized as follows: Section 2 reviews related works compared with our scheme. Preliminary knowledge and research motivation are given in Section 3. Section 4 describes the network model and defines problem statements of this paper. In Section 5, we give the design of Cross-layer Optimized Opportunistic Routing (COOR) scheme for loss-and-delay sensitive WSNs. Section 6 proposes experimental results and comparison of COOR scheme. We conclude this paper in Section 7.

## 2. Related Work

### 2.1. Loss Sensitive Works in WSN

This section focuses on the reliability and delay of data transmission in wireless sensor networks [11,29,35,37,47]. Due to interference, low power transmission, conflicts and other factors, wireless communication is error-prone and unreliable [50,51]. Aiming at maintaining the reliability of communication, there are many studies on this topic, which mainly concentrate on the network routing layer, so we first introduce the transmission reliability guarantee mechanism of the wireless sensor network routing layer:

(1) An opportunistic routing scheme [49] serves as a basis for this paper. According to previous research [47], one-hop transmission reliability cannot be expected in wireless sensor networks. Sometimes the packet error rates (PER) of the communication links between two sensor nodes are more than 10% [47]. In this case, without adopted of a PER 10% (i.e., reliability = 90%) reliability transmission mechanism in the network, the probability of successfully reaching a sink at 10 hops is only 34.86%. Worse still, if the route hops are greater, the receiving success rate will be too low to meet the requirements of the application. Therefore, a reliable one-hop transmission is indispensable in the improvement of the end-to-end reliability.

As for opportunistic routing, it improves the reliability of data transmission by the following methods [49]: first, the sender determines a candidate set which comes from the Forwarding Nodes Set (FNS). The forwarding node set is a collection of nodes that are within the sender’s transmission distance, but closer to the sink than the sender. The candidate set consists of those nodes in the FNS that are close to the sink and have high reliability. The sender transmits information forward by broadcasting to all nodes in its candidate set. The transmission succeeds as long as any node in the candidate set receives the message correctly. If there is more than one node accomplishing this, the most favorable node to transmit to the sink will continue routing, while the others will simply drop their received messages. At the same time, in the transmission process, the data only needs to be sent once, and received by multiple nodes. Compared with unicast transmission methods, in which many receiving nodes imply multiple sending times, the energy consumption of opportunistic routing is rather economical. In addition, its delay is relatively small, mainly because it takes less time to transfer data, though, determining which is the most favorable node to continue forward routing is requires communication between candidate nodes, and this will increase the delay slightly.

(2) Retransmission mechanism, one of the most widely used reliability assurance mechanisms [47], is characterized by simple requirements and wide application. Automatic Repeat-reQuest (ARQ) is the most basic protocol of this mechanism, and its principle is as follows: when a sender node finishes sending a packet, it starts a timer and waits for the receiver to confirm the information. Once the packet is received correctly, the receiver returns an ACK message. Then, if the sender node receives the ACK message in time, it will consider that the previous data packet has been received successfully, so the next packet can be sent, but if the sender has still not received the ACK message after waiting beyond the scheduled time, the packet is considered to have been lost and needs to be sent again. The retransmission will be repeated until the sender receives the ACK message or reaches a threshold time. At that point, the transmission of the current packet is considered to be aborted, and the next packet is sent instead.

Thus, the ARQ protocol can effectively maintain reliable data transmissions without high energy consumption. In detail, the additional energy consumption only comes from ACK messages, which cost is rather limited due to its small length. However, there is a drawback of high delay. Especially when the data packets is lost during delivery, the sender needs to wait for a relatively long round trip time (RTT) before each retransmission. Furthermore, a send-and-wait (SW) protocol is an important mechanism to protect the reliability of data transmission [47]. In subsequent studies a variety of improved methods are proposed, such as the Goback-N (GBN) protocol, or the selective repeat (SR) [47] algorithm. Specifically, a strategy for high energy efficiency of wireless sensor networks to ensure data transmission reliability can be seen in [47].

(3) Data collection by broadcasting is an effective way to improve data transmission reliability. Joo and Shroff [52] proposed a method of broadcast data transmission in a fusion network. In the network of [52], the data of an infinite number of nodes can be merged into one data packet when they meet, so the amount of data that the network needs to transmit is hardly increased. Thus, in the method of [52], each node routes during broadcast, and the receiver node collects data packets from all child nodes and merges them into one packet to continue broadcasting to the sink. Since the fused data packet contains all the information of the received data packets, it is equivalent to broadcasting each data packet and routing multiple copies to the sink along different paths. However, the biggest limitation of this method is that it requires multiple data packets be fused into one packet. Otherwise, a broadcast storm will be formed and the network energy will be quickly consumed. On this basis, for a more general data fusion network readers can refer to [29].

(4) Improve the reliability of data transmission by multi-layer optimization in [29]. The reliability of data transmission is directly determined by the signal-to-noise ratio (SNR). With the increase of the signal-to-noise ratio of the receiver, the probability of correctly receiving data increases and the bit error rate (BER) decreases. Although there are many factors that influence signal-to-noise ratio, the external environment of sensor nodes is not selectable after deployment, so the most effective way to improve SNR is to increase the transmission power of the sender. Unfortunately, with very limited energy of the wireless sensor node, increasing the transmission power will seriously affect the network lifetime. In wireless communication, a relatively low SNR means a quite unreliable communication link, but as long as the transmission power is slightly increased, the SNR will be promoted distinctly, and the success rate of data transmission will rise significantly. However, when the data transmission success rate reaches a certain value, even if the transmission power increases multiply (the lifetime reduces multiply), the improvement of the data transmission success rate is still rather small. It can be seen that instead of improving the transmission power of node alone, adopting multiple methods is more likely to achieve good performance, such as cross-layer optimization combined with the network layer [35]. This is also the starting point of this paper.

Considering the nonlinear relationship between SNR and data receiving rate, another way to improve the reliability of data transmission in low power condition is by reducing the distance between sender and receiver. In wireless communication, the speed of signal fading is proportional to 2 or even 4 times the distance. Therefore, short-distance multi-hop transmission is more appropriate for wireless sensor networks. According to this principle, the optimization of data transmission can be achieved by controlling the distance between nodes during network deployment. Meanwhile it is easier to balance the energy consumption and reliability of data transmission by this method.

### 2.2. Delay Sensitive Works in WSN

In contrast, ensuring reliable data transmission is the main research content, and delay mainly depends on the reliability guarantee mechanism. Some delay-sensitive works of wireless sensor network routing layer are expressed as follows:

(1) Coding-based reliability guarantee mechanism [53,54]. According to the mechanism of redundancy encoding, the data packet is encoded before being sent. With the help of redundant code, a receiver can still decode the received data packet to get the correct information, even if there losses in wireless communication due to some errors.

Reed-Solomon coding is an effective redundancy method [53,54]. There have been some studies on the application of Reed-Solomon coding to reliable data transmission in wireless sensor networks. In [53], a Reed-Solomon codec algorithm for WSNs is proposed to reduce energy consumption. In [54] packets are encoded the by Reed-Solomon code and routed to the sink node along multiple paths. Network coding is regard as an effective data transmission scheme. Ahlswede and others [55] first proposed the concept of network coding in 2000. The basic idea is that the network nodes can encode multiple packets from different links (for example, through XOR or linear coding, etc.) into one packet, and then send it out. As a result, the amount of information in a single transmission is increased exponentially.

The advantage of reliable data transmission based on coding techniques is that sender can transmit data to receiver at a high probability of success with only one attempt. Therefore, it has a small delay for saving the cost caused by multiple retransmission. The disadvantage, however, is that the coding scheme requires the data packets append extra information for correction, but the length of the additional encoded data is not negligible. As a consequence, the amount of data is increased, and the nodes need to encode and decode, both of which require a certain processing time and will increase the load of the nodes. Simultaneously, the communication capability of the network is actually reduced as the probability of data transmission conflict is increased, and the network lifetime is reduced.

(2) Data fusion can also be considered as a coding mechanism. For example, in the application of querying the average value, the maximum value, and the minimum value in the network, multiple data packets pass through one node. After the calculation (equivalent to encoding) of all received packets, a node will fuse this information into one data packet and continue routing. Hence, this method can effectively reduce the amount of data and the transmission conflicts, and prolong the network life, so it is widely used in all kinds of applications [56]. Certainly, in wireless sensor networks data fusion is most often used when multiple data packets can be gathered and generalized to be smaller. Related studies can be found in [56].

Therefore, the general research on reliable data transmission will also analyze the delay of the method adopted. In the same way as previous researches, this paper first proposes a data reliability transmission scheme, and then analyzes the delay performance. Similarly, this paper only considers the transmission reliability and delay [57] caused by the quality of the communication link, but irrespective of the impact of network security problems [58,59,60].

## 3. Preliminary Knowledge and Research Motivation

### 3.1. Details of Opportunistic Routing

As a basis for this paper, more detailed features of opportunistic routing are explained in this section. The progress of opportunistic routing are as follows: before sending a packet, a node first selects a candidate set G based on local information, and then broadcasts the packet to all candidate nodes. When the transmission is accomplished, the candidate set G determines an optimal node i to be the current forwarding node according to some specific metrics. After reaching an agreement, the optimal node i continues routing, while the other nodes in the collection G delete their local packets. The above steps are repeat until the destination node receives the packet.

**Figure 1:**
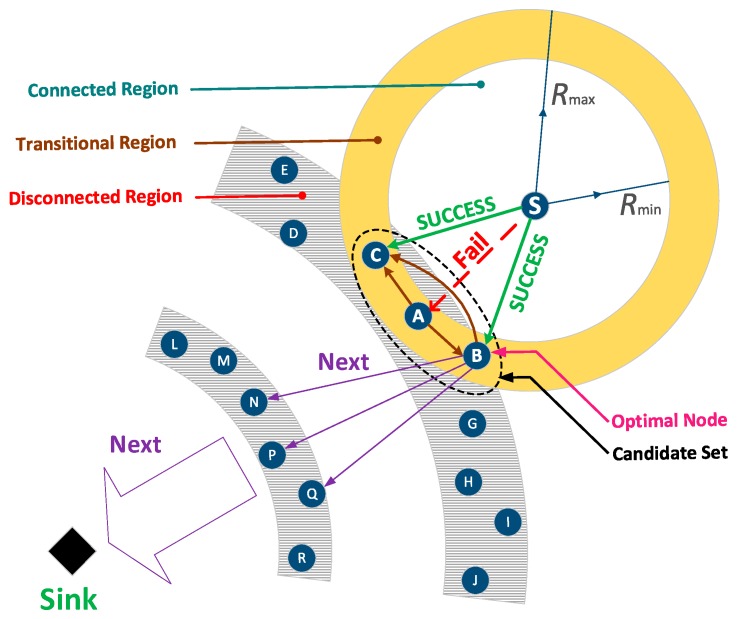
Illustration of opportunistic routing scheme.

As shown in the Figure 1, the node S is the source node that need to route packets to the sink. According to the reliability model, the transmission range of node S is divided into three areas [56]: (1) Connected region. Nodes in this area are close to the node S, so the reliability of transmission between them is high. (2) Disconnected region. Far from the node S, nodes in this domain have a rather low success rate of receiving packets from the node. (3) Transition area, where nodes have a specific range of success rates. If the node in the connected region is selected as the receiving node, the communication link is reliable, but the forward distance per hop is small. If we turn to the disconnected area, the reception rate is rather low. Therefore, the opportunistic routing selects multiple nodes in the transition region and broadcasts. In this way, unless all chosen nodes fail to receive data, the current transmission is successful. Obviously, it is an efficient routing method with high reliability, and maintains a certain distance per hop.

Most research [29] results show that the appropriate regional division is based on the quality of communication link between nodes. If the Packet Reception Rate (*PRR*) is less than 0.1, the node belongs to a disconnected area. Otherwise, if the *PRR* of the transition area is between 0.1 and 0.9, and the *PRR* of the connected area is greater than 0.9.

For instance, node A, B and C are the three candidate nodes selected by node S in its transition region. As can be seen in Figure 1, node A fails to receive data, while node B and node C succeed. Then, the candidate nodes sequentially confirm the reception status according to the priority order. First, Node A sends a Not Acknowledge (NAK) packet to node B and node C, informing them that it has failed to receive the current data packet. Then, the next priority node B checks and sends an ACK packet to inform node C and the sending node S that its reception is successful. Finally, node C deletes the current local packet and one-hop transmission is successfully finished. This process is repeated until the packet reaching the sink.

Assuming the number of candidate nodes is N and the transmission success rate between the send node and the receive node is p, the one-hop reliability can be computed as:

(1) $$\zeta =1-{\left(1-p\right)}^{N}$$

Furthermore, if the probability that any node in the A, B and C successfully receives a packet from node S is equal to 0.7, the traditional routing method chooses one of them as the forwarding node directly, which renders that the one-hop reliability ζ is equal to 0.7. In the same situation, however, the one-hop reliability of the opportunistic routing is $\zeta =1-{\left(1-0.7\right)}^{3}=0.973$. From this, it can be seen that the opportunistic routing can improve the transmission reliability to a certain extent. The more the candidate nodes selected, the more obvious the improvement of the reliability.

### 3.2. Research Motivation

Maintaining a reliable and low delay data transmission in wireless sensor networks is achieved by multiple factors. It is not only affected by the network routing layer, but also related to the sending power. Therefore, in order to effectively improve the network performance, a cross-layer optimization method is proposed in this paper. The research motivation is illustrated through the following experiments. The values of wireless sensor network parameters are given collectively in Table 1.

**Figure 2:**
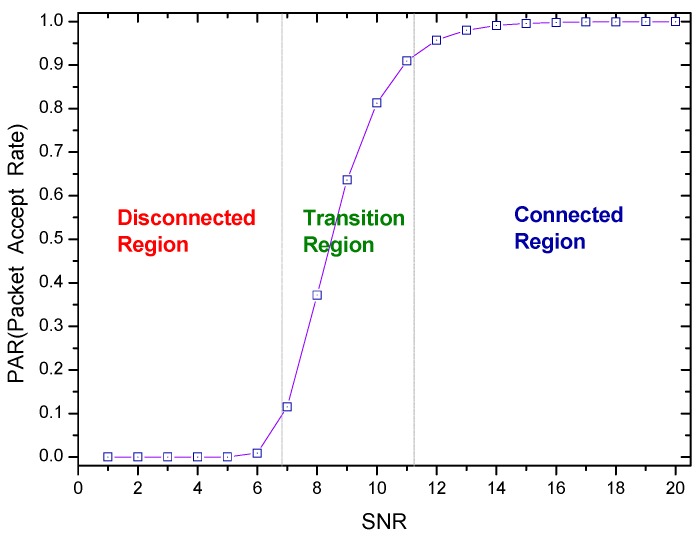
Packet acceptance rate and signal-to-noise ratio.

The Packet Acceptance Rate (*PAR*) and *SNR* have a progressive correlation (which can be seen from Figure 2) and the greater signal-to-noise ratio, the higher data packet acceptance rate.

**Figure 3:**
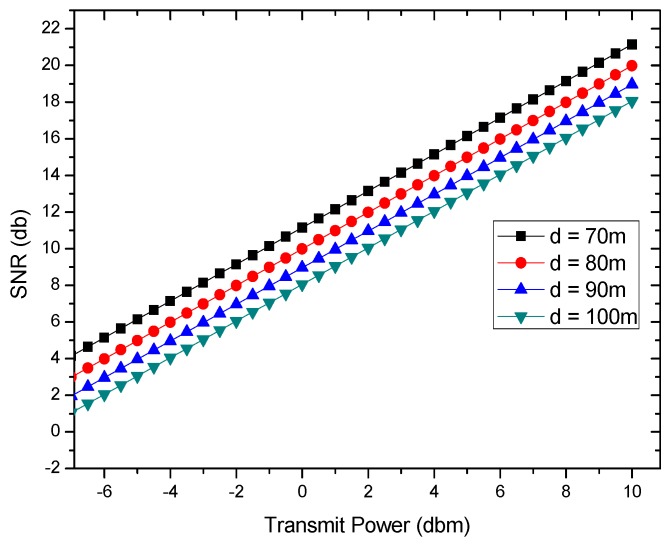
Signal-to-noise ratio in different transmit power with different distance.

Figure 3 further shows the relationship between *SNR* and transmit power Pt with different distance d. Obviously improving the *SNR* requires increasing the transmission power or reducing the transmission distance.

**Figure 4:**
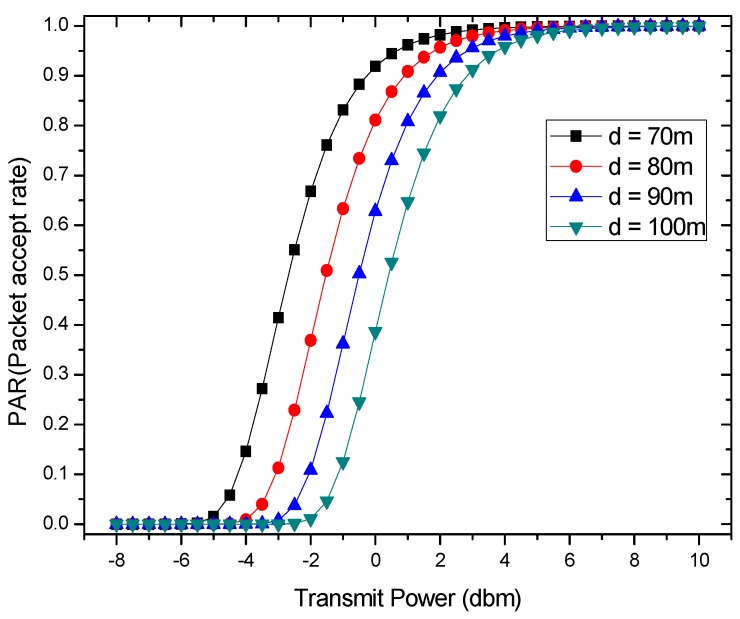
Packet acceptance rate and transmit power.

**Figure 5:**
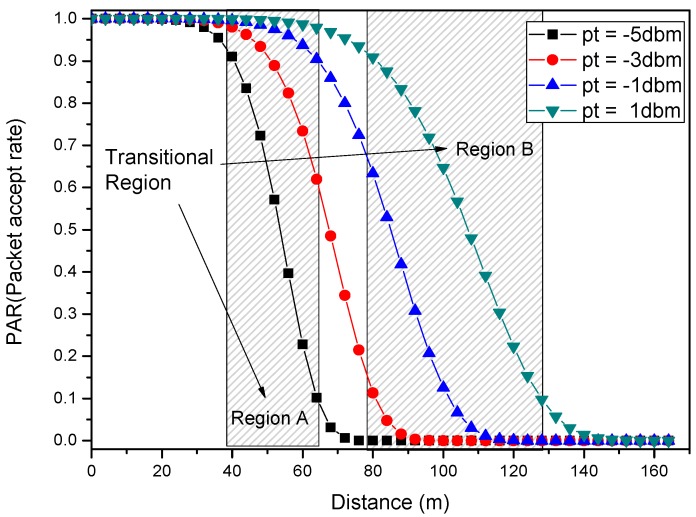
Packet acceptance rate and transmission distance.

Figure 4 and Figure 5 respectively show the relationship between *PAR* and transmission power Pt as well as distance d. The *PAR* will improve with the increase of the transmission power Pt. Its growth relationship can be divided into three segments. It can be seen that the value of *PAR* is kept at 0 until it reaches a certain value and starts to grow rapidly, and finally it stays almost constant after reaching 1. In contrast, the *PAR* decreases with the longer transmission distance d. The descent relationship can also be divided into three similar segments (which can be seen in Figure 5).

From the information in Figure 5, the impact of increasing the transmission power on the transition region can be generated as a farther and larger transition area with same one-hop reliability. If the transition region is fixed, the same communication link will become more reliable. However, increasing the transmission power requires more energy consumption. Unfortunately, the energy of a node in wireless sensor network is limited, so the improvement of transmission power is limited. After many experiments, we find that although the energy of wireless sensor networks is limited, the energy consumption of each node is very uneven.

**Figure 6:**
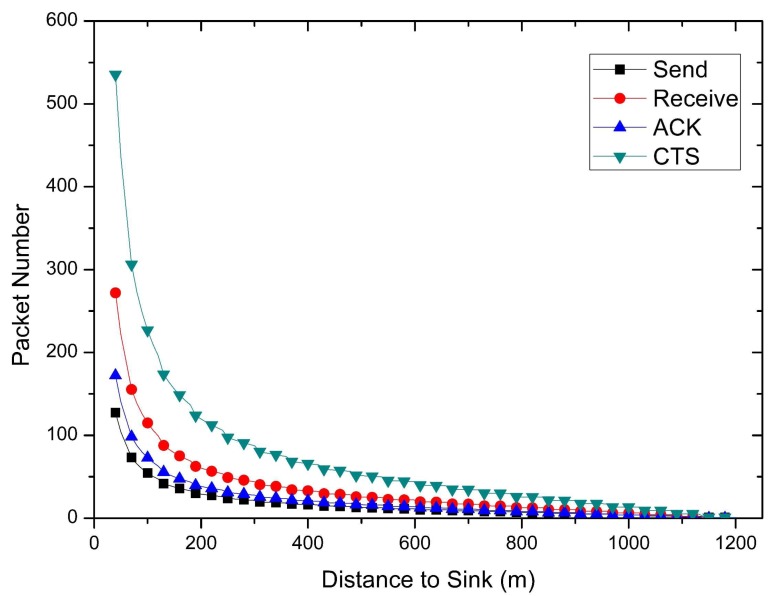
Packet load in different areas.

**Figure 7:**
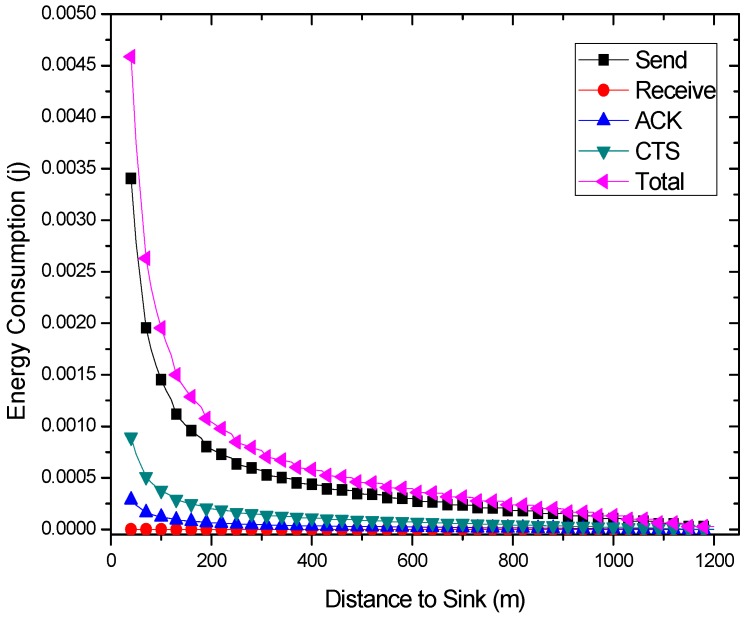
Energy consumption in different areas.

As shown in Figure 6, since the nodes in the near-sink area need to relay the data packets from the nodes in far-sink area, the data volume of near-sink nodes is larger. This results in a very high energy consumption of the nodes near the sink, and a large amount of energy remaining in the nodes far away from the sink (which can be seen in Figure 7). Network performance can be improved by taking full advantage of the remaining energy. Based on these circumstances, two different methods to improve WSNS performance in COOR strategy are proposed in this paper.

COOR(R) method: This keeps the transmission radius of each hop constant but improves the transmission power of each node in the far-sink region to a certain extent according to its energy surplus, which is related to the distance to the sink. Directly, the transmission reliability is improved greatly. As a consequence, the time for communication and confirmation between candidate nodes can be reduced, which can slightly reduce the delay of the whole system.

COOR(P) method: This increases both the transmission radius and the transmission power of each node in the far-sink region to a certain extent, but maintains the *PAR* of each hop unchanged. As the transmission radius of each node increases, the number of hops to the sink decreases but the distance to the sink remains same. Therefore, the goal of reducing delay and improving network reliability is achieved.

**Figure 8:**
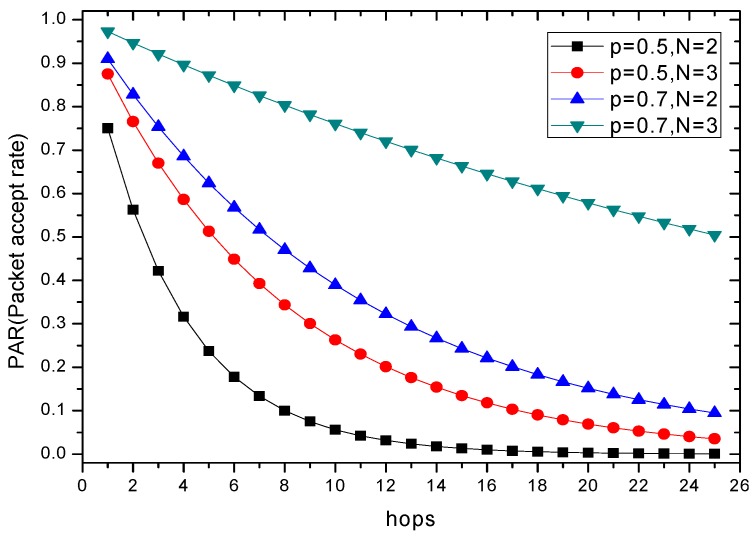
End-to-end reliability in opportunistic routing.

Examples to illustrate how the COOR method can improve network reliability follow. Consider the same reliability of one-hop transmission, the relation between the end-to-end reliability and the number of routing hops are shown in Figure 8. Furthermore, the influence of single transmission success rate p with different size N of candidate nodes set can also be obtained. End-to-end reliability will gradually decrease as the number of hops increases. Simultaneously, both the reliability of single transmission and the size of candidate set have a positive promotion on end-to-end reliability.

Accordingly, the COOR(R) method keeps hops invariant and improves p, while the COOR(P) method maintains the p unchanged and reduces hops. Therefore, the two methods increase the reliability of the WSNs from different perspectives. At the same time, it is obvious that if the transmission power of the node is increased too much, the energy consumption of node increases significantly, which will seriously shrink the network life, so how to calculate the optimal transmission power to fully utilize the remaining energy to enhance network performance, the core problem of the COOR scheme, will be explained in the next section.

## 4. System Model

### 4.1. Network Model

We consider a periodic data collection wireless sensor network [29]. In this network, a large number of sensor nodes are deployed randomly with density ρ in a circular area with a radius 𝑅 to monitor continuously some preset activities. All the sensor nodes are same in the aspect of initial energy, and sending rate. Like in [61,62], an opportunistic multi-hops routing protocol is used.

A special node called sink locates at the center of the network, which is set for collecting the data generated by the entire network to achieve some specified function. Each sensor node monitors a nearby environment and generates a data packet in a sensing period. All the data packets will be sent directly or transmit by relay nodes in a multi-hop style to the sink unless losses or errors happen.

In addition, we assume the MACA protocol being included in MAC layer to mitigate the hidden station problem and exposed station problem [62].

A successful transmit is performed if both data and ACK packets are received without errors by the intended recipients [62]. If either of them has an exception, the transmission is regarded as failed. When a failure appears, retransmission will not be performed. The sender node will reorganize the data packet and transmit it in the next round.

### 4.2. Reliability Model

Same as the reliability model proposed by Joo [56], communication between nodes can cause errors. Consider the communication link between two nodes having a distance separation of d. The propagation path loss can be expressed as [56]:

(2) $$PL\left(d\right)=\;PL\left(d_{0}\right)+10n\cdot \log_{10}\left(\frac{d}{d_{0}}\right)+X_{\sigma }$$

Here, $d_{0}$ is a reference distance and $PL\left(d_{0}\right)$ represents the path loss at that distance. n is the path-loss exponent; $X_{\sigma }$ is the shadowing component Obeying the Gauss distribution with zero mean and standard deviation. In most situations, $X_{\sigma }$ is a random process, which means a function of time. Since not assuming the dynamic environment, we use it as a constant random variable to model a specific link with time.

Considering a transmit power of $P_{t}$ and an additive white Gaussian noise (AWGN) power of $P_{n}$, the *SNR* at the receiver is obtained as [56]:

(3) $$\gamma {\left(d\right)}_{dB}=\;P_{tdB}-PL{\left(d\right)}_{dB}-P_{n}{}_{dB}$$

According to [29], $P_{n}$ depends on wireless signal and environment, and its value is given [56]:

(4) $$P_{n}=\left(F+1\right)kT_{0}B$$

Here, F is the noise factor, k is the Boltzmann constant, $T_{0}$ is the environment temperature, and B is the equivalent bandwidth. According to [61], the common network environment have a background noise values −115 dBm.

For the reliability performance evaluation, we adopt the communication link model reported in [29]. In this paper, the Packet Acceptance Rate (*PAR*) is used to measure the quality of the communication link, and it can be calculated by the following equation [29]:

(5) $$par_{\left(d\right)}={\left(1-\frac{1}{2}exp^{-\;\frac{SNR_{\left(d\right)}}{2}\times \frac{B_{N}}{R_{D}}}\right)}^{8f}$$

Here, d is the node spacing, $R_{D}$ is the data rate, f is the size of a data packet (in bytes), and $B_{N}$ is noise bandwidth.

According to [47], the end-to-end reliability ψ is the product of all the one-hop transmission reliability on the routing path, which can specifically be expressed as:

(6) $$\psi =\zeta_{1}\cdot \zeta_{2}\cdot \zeta_{3}\cdot \cdots \cdot \zeta_{k}$$

where $\zeta_{i}$ is the reliability of the $i^{th}$ hop.

### 4.3. Energy Consumption Model

Similar to the most energy consumption models for WSNs [56], the energy consumption depends on the transmission power and the time duration of frame transmission and reception. At the same time, the energy consumption of the nodes mainly comes from sending and receiving packets [29], sending ACK packets, and sending Clear-To-Send (CTS) communication control packets. Other energy consumption is negligible compared to these. In general, the energy consumption of a node to transmit a packet can be obtained by multiplying the transmission power of the current node by the transmission time. Meanwhile, the transmission time can be calculated by dividing the data volume by the data rate.

The energy consumption of sending data packets is:

(7) $${Ε}^{s}=P_{t}\cdot L^{s}\cdot Q^{S}/R_{D}$$

The energy consumption of receiving data packets is:

(8) $${Ε}^{r}=P_{r}\cdot L^{s}\cdot Q^{R}/R_{D}$$

The energy consumption of sending CTS packets is:

(9) $${Ε}^{CTS}=P_{t}\cdot L^{CTS}\cdot Q^{C}/R_{D}$$

The energy consumption of sending ACK packets is:

(10) $${Ε}^{ACK}=P_{t}\cdot L^{ACK}\cdot Q^{A}/R_{D}$$

The total energy consumption of the current node is:

(11) $${Ε}^{TOT}=\;{Ε}^{s}+{Ε}^{r}+{Ε}^{CTS}+{Ε}^{ACK}$$

According to [59], the receiving power of the current node is given as:

(12) $$P_{r}=P_{t}-PL\left(d\right)$$

Here, $R_{D}$ is the data rate (in bits/s). $Q^{S}$ is the data volume of the current node to send and $Q^{R}$ is the data volume of the current node to receive. $Q^{CTS}$ is the total number of the CTS packets sent by the current node and $Q^{ACK}$ is the total number of the ACK packets sent by the current node.

$L^{s}$ is the length of a data packet (in bits). $L^{CTS}$ and $L^{ACK}$ respectively represents the length of a CTS packet and an ACK packet (in bits).

### 4.4. Problem Statement

The study of Cross-layer optimized Opportunistic Routing (COOR) is a multiple target optimization problem. The goal in this paper is to maximize the network lifetime to minimize the probability of packet loss and delay. Just like in [29], the Cross-layer optimization of a WSN can be characterized by several performance indicators as explained below:

(1) End-to-sink delay ($T_{sink}^{x}$). End-to-sink delay refers to the period from the moment a node with distance x m to sink starts sending a packet until the sink finally receives it successfully. $T_{sink}^{x}=\sum_{i=0}^{k}T_{i}^{x}$, where k indicates the hops during the entire transmission and $T_{i}^{x}$ refers to the time of the $i^{th}$ hop. Obviously, the lower the end-to-sink delay the better, which can be expressed as:

(13) $$min\left(T_{sink}^{x}\right)=min\left(\overset{k}{\underset{i=1}{\sum }}T_{i}^{x}\right)$$

(2) Packet transmission reliability ($P_{sink}^{x}$). Packet transmission reliability refers to the probability that the packet send by a node with distance x m can be received by the sink. Clearly, the higher the $P_{sink}^{x}$ the more reliable of the network:

(14) $$\{\begin{array}{c} max(P_{sink}^{x})=\overset{k}{\underset{i=1}{\prod }}par_{\left(d_{i}\right)}\\ s.t.\;x\leq \overset{k}{\underset{i=1}{\sum }}d_{i} \end{array}$$

Here, k has the same meaning as mentioned above. Meanwhile $d_{i}$ and $par_{\left(d_{i}\right)}$ indicate the distance and the success rate of the ith transmission.

(3) Network lifetime (L). Like Reference [29], lifetime is defined as the death time of the first node in the network. Here, we assume the initial energy of node with distance x being $E_{INT}^{x}$ and the energy consumption being $\varepsilon_{x}$ per unit time. In conclusion, the lifetime is then given by the following equation:

(15) $$max\left(L\right)=max\left(\min\left(\frac{E_{INT}^{x}}{\varepsilon_{x}}\right)\right)$$

Because of the death time of the first node is defined as network lifetime, minimizing the energy consumption of the node spends the most is equivalent to maximize the lifetime. Therefore Equation (15) can also be expressed as:

(16) $$max\left(L\right)=\min(\max\left(E_{i}^{TOT}\right))$$

Obviously, the goal of Cross-layer optimization can be stated as follow Equation (17):

(17) $$\{\begin{array}{c} min\left(T_{sink}^{x}\right)=min\left(\overset{k}{\underset{i=1}{\sum }}T_{i}^{x}\right)\\ max(P_{sink}^{x})=\overset{k}{\underset{i=1}{\prod }}par_{\left(d_{i}\right)}\\ max\left(L\right)=\min(\max\left(E_{i}^{TOT}\right))\\ s.t.\;T_{sink}^{x}\leq T^{\theta },\;P_{sink}^{x}\geq P^{\theta },L\geq L^{\theta },x\leq \overset{k}{\underset{i=1}{\sum }}d_{i} \end{array}$$

Here, $T^{\theta },P^{\theta },L^{\theta }$ represent the minimum requirements of the transmission performance thresholds in applications. The goal of Equation (16) is to minimize the transmit performance $T_{sink}^{i}$, and keep it not less than the minimum requirements of the application performance. Meanwhile, make the network lifetime and reliability maximized.

## 5. The Design of the COOR Scheme

### 5.1. Transmission Power Optimization

The COOR scheme is the improvement of the basic network model. Thus, it is necessary to calculate the data volume and the energy consumption of each tier in the wireless sensor network under constant power. Then, according to this information, the node transmission power of different areas is adjusted to achieve the purpose of optimization.

The calculation of data volume is to count the number of data packets that each node should bear. The exact number of packets is critical in computing the energy consumption. Therefore, the following describes how to calculate the number of packets that each node bears.

**Figure 9:**
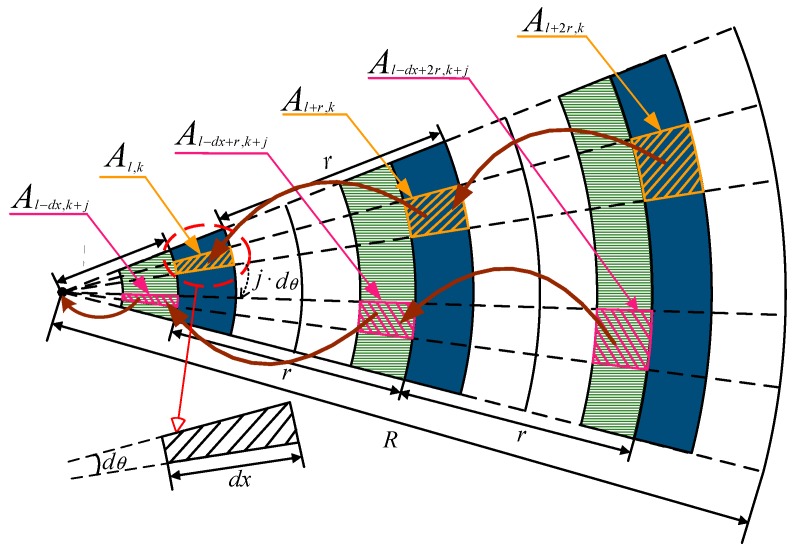
Network transmission structure.

The basic network transmission structure is given in Figure 9. The radius of the circular network is R, and the node transmission radius is r. The nodes in the network are evenly distributed and the density is ρ. The area $A_{l,k}$ is a fan-shaped ring area with a distance l to the sink, a width $d_{x}$, and a radians value $d_{\theta }$. The area $A_{l+x,k+j}$ is x far from $A_{l,k}$ with the difference in the clockwise radians $j\cdot d_{\theta }$.

**Theorem** **1.** *Consider a small fan-shaped ring region* $A_{l,k}$ *, the total number of nodes contained in this area is as:*

(18) $$S_{l,k}=\rho ld_{\theta }d_{x}$$

**Proof.**  As shown in Figure 9, the area of region $A_{l,k}$ can be calculated by the following formula:

$$s=\frac{1}{2}{\left(l+d_{x}\right)}^{2}d_{\theta }-\frac{1}{2}l^{2}d_{\theta }$$

The number of nodes is equal to the distribution density multiplied by the area:

$$S_{l,k}=\rho s=\rho ld_{\theta }d_{x}$$

□

Further analysis of the data volume of a specific node in different regions is as follows: consider the edge area of the network (R−r<l≤R), the nodes in this part of the network do not undertake the forwarding of packets from any other node, so the number of packets sent is 1, the number of packets received and the number of ACK packets sent are 0. At the same time, the number of CTS packets it sends is 1, which is used to confirm the communication clear before the next hop node replies ACK packet.

**Figure 10:**
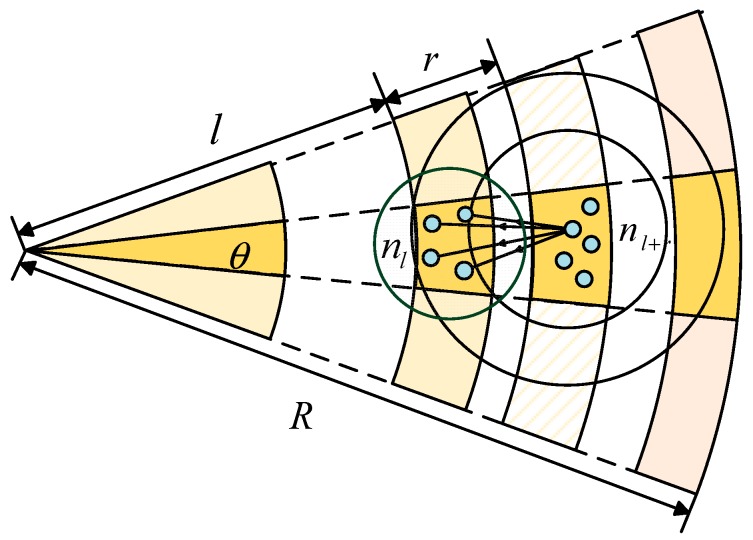
Calculation diagram of data volume.

For non-edge areas in the network, as shown in Figure 10, region $A_{l,k}$ will rely all packets sent from $A_{l+r,k}$. If the partition is small enough, then for any node $n_{l+r}$ in the region $A_{l+r,k}$, it is the same to choose any node $n_{l}$ in $A_{l,k}$ in the aspect of transmission reliability.

**Theorem** **2.** *When the success rate of a single transmission between node*$n_{l+r}$*and node*$n_{l}$*is*$p_{l+r}$, *in a round of sending each node sends a packet to the sink. Then, respectively, the number of sending packets*, *in a round of sending each node sends a packet to the sink. Then, respectively, the number of sending packets*$Q^{S}\left(l\right)$*, receivedpackets*$Q^{R}\left(l\right)$*, ACK packets*$Q^{A}\left(l\right)$*and CTS packets*$Q^{C}\left(l\right)$*of node*$n_{l}$*can be calculated as:*

(19) $$Q^{S}\left(l\right)=\;\frac{l+r}{l}\cdot \left(1-{\left(1-p_{l+r}\right)}^{N}\right)\cdot Q^{S}\left(l+r\right)+1$$

(20) $$Q^{R}\left(l\right)=\;\frac{l+r}{l}\cdot N\cdot Q^{S}\left(l+r\right)$$

(21) $$Q^{C}\left(l\right)=Q^{S}\left(l\right)+Q^{R}\left(l\right)\left(1+\frac{1}{p_{l+r}}-\frac{1-{\left(1-p_{l+r}\right)}^{N}}{Np_{l+r}{}^{2}}\right)$$

(22) $$Q^{A}\left(l\right)=Q^{R}\left(l\right)\cdot \frac{1-{\left(1-p_{l+r}\right)}^{N}}{Np_{l+r}}$$

**Proof.**  All the nodes under the traditional routing method have the same transmission radius, so the transmission between the areas is periodic. As shown in Figure 9, region $A_{l,k}$ assumes the forwarding of the data generated by the nodes in region $A_{l+r,k},A_{l+2r,k},\cdots A_{l+mr,k}$, where $m=\lfloor \frac{R-l}{r}\rfloor$.

For the area $A_{l,k}$, all the nodes in this area directly and uniformly assume the forwarding of data packets sent by the nodes in the outer area $A_{l+r,k}$ with distance r. According to Equation (18), there are a total of $\rho \left(l+r\right)d_{\theta }d_{x}$ nodes in the region $A_{l+r,k}$, and each node will send packets $Q^{S}\left(l+r\right)$. At the same time, each data packet is received N times and each time it is successfully received with probability $p_{l+r}$.

Therefore, the total number of packets received by the region $A_{l,k}$ is $\rho \left(l+r\right)d_{\theta }d_{x}\cdot Q^{S}\left(l+r\right)\cdot N$, averaging to $\rho ld_{\theta }d_{x}$ nodes, and the number of received packets of node $n_{l}$ can be obtained by:

$$Q^{R}\left(l\right)=\frac{l+r}{l}\cdot N\cdot Q^{S}\left(l+r\right)$$

The data packets sent by node $n_{l}$ are divided into two parts: (1) Self-generated (2) Assist the outer node to forward. Because it is a round of sending time, the number of data packets generated by each node is always 1. In the second case, the total number of packets to be forwarded is $\rho \left(l+r\right)d_{\theta }d_{x}\cdot Q^{S}\left(l+r\right)$, and each packet is successfully received by the region $A_{l,k}$ at the probability of ${\left(1-p_{l+r}\right)}^{N}$. Consequently, the average number of data packets sent by node $n_{l}$ in a round of sending is:

$$Q^{S}\left(l\right)=\;\frac{l+r}{l}\cdot \left(1-{\left(1-p_{l+r}\right)}^{N}\right)\cdot Q^{S}\left(l+r\right)+1$$

The node $n_{l}$ sends CTS messages mainly considering three kinds of situations: (1) Replying to the last hop node to receive data. (2) Preparing to receive communication information of the node in the same area $A_{l,k}$. (3) Preparing to receive the ACK messages sent by the next hop node.

In the first circumstance, the number of CTS packets to be sent is equal to the number of packets it receives. As for the third circumstance, the number of CTS packets in this part is the same as the data volume of the current node to send.

The second circumstance is a little more complicated for it is related to the priority of current node $n_{l}$ in the candidate set. In the basic network, since the performance and status of each node in the region $A_{l,k}$ are regarded as same, it can be considered that the probability at each priority in each transmission is the same (1/N). When the node is at the ith priority in current transmission, it needs to consider the reception condition of the first (i−1)th priority nodes in turn. Specifically, the communication between candidate nodes won’t stop until one of them claim its successful reception. In this condition (the ith priority), the CTS packets node $n_{l}$ sends is expressed as:

(23) $$Q_{l}^{i}=\overset{i-1}{\underset{j=1}{\sum }}j\cdot p_{l+r}{\left(1-p_{l+r}\right)}^{j-1}+\left(i-1\right){\left(1-p_{l+r}\right)}^{i-1}$$

The jth term here indicates that the nodes of the previous (j−1)th priority all failed to receive, and the jth priority node succeeded. Generally, the probability of its occurrence is $p_{l+r}{\left(1-p_{l+r}\right)}^{j-1}$, and there are j CTS packets that the node needs to send (response *j* times). While if the first i−1 priority nodes all fail to receive, then the node $n_{l}$ will send a total of *i* − 1 CTS packets (in response to the previous *i* − 1 nodes).

The following is a simplification of the expression (23):

$$Q_{l}^{i}=\left(i-1\right){\left(1-p_{l+r}\right)}^{i-1}+p_{l+r}\overset{i-1}{\underset{j=1}{\sum }}j\cdot {\left(1-p_{l+r}\right)}^{j-1}$$

Assume that:

$$S_{l}^{n}=\overset{n}{\underset{j=1}{\sum }}j\cdot {\left(1-p_{l+r}\right)}^{j-1}$$

Obviously, it can be obtained:

$$\left(1-p_{l+r}\right)\;S_{l}^{n}=\overset{n}{\underset{j=1}{\sum }}j\cdot {\left(1-p_{l+r}\right)}^{j}$$

The subtraction of two equations above:

$$p_{l+r}S_{l}^{n}=-n{\left(1-p_{l+r}\right)}^{n}+\overset{n}{\underset{j=1}{\sum }}{\left(1-p_{l+r}\right)}^{j}$$

Plugging n=i−1 into the Equation (23):

$$Q_{l}^{i}=\left(i-1\right){\left(1-p_{l+r}\right)}^{i-1}+p_{l+r}S_{i-1}$$

$$Q_{l}^{i}=\overset{i-1}{\underset{j=1}{\sum }}{\left(1-p_{l+r}\right)}^{j}$$

The result can be given by using the sum formula of the geometric progression:

$$Q_{l}^{i}=\frac{1-{\left(1-p_{l+r}\right)}^{i-1}}{p_{l+r}}$$

In summary, the total number of CTS packets sent by a node can be obtained:

$$Q^{C}\left(l\right)=Q^{S}\left(l\right)+Q^{R}\left(l\right)\cdot (1+\frac{1}{N}\overset{N}{\underset{i=1}{\sum }}\;Q_{l}^{i})$$

$$Q^{C}\left(l\right)=Q^{S}\left(l\right)+Q^{R}\left(l\right)\left(1+\frac{1}{p_{l+r}}-\frac{1-{\left(1-p_{l+r}\right)}^{N}}{Np_{l+r}{}^{2}}\right)$$

The ACK messages sent by node $n_{l}$ are mainly used to confirm whether it has successfully received the data packets sent by the last hop to the other candidate nodes with lower priority in a single transmission. Meanwhile, it is also used to inform the sending node of the final receiving result of the current packet in this hop transmission.

In a single transmission, each candidate node sends an ACK message in order of priority, and if a candidate node with a priority greater than the current node confirms that its recipient succeeds, the current node deletes the same packet immediately. This means that a single-hop transmission is finished, and the current node does not need to send an ACK message. Therefore the total number of ACK messages sent by the node $n_{l}$ in a round is given as:

$$Q^{A}\left(l\right)=Q^{R}\left(l\right)\cdot \frac{1}{N}\cdot \overset{N-1}{\underset{i=0}{\sum }}{\left(1-p_{l+r}\right)}^{i}$$

$$Q^{A}\left(l\right)=Q^{R}\left(l\right)\cdot \frac{1-{\left(1-p_{l+r}\right)}^{N}}{Np_{l+r}}$$

□

For traditional routing methods, typically, all nodes not only have the same emission distance but also have the same transmit power. According to Equations (2) and (4), the success rate of single transmission $p_{l}$ is equal everywhere in the network. Given these circumstances, a special case of Theorem 3 can be deduced:

**Theorem** **3.** *Considering that the success rate of a single transmission in the traditional routing method is* p *. The numbers of all kinds of packets sent or received by the node* $n_{l}$ *in a round of transmission are as follows:*

(24) $$Q^{S}\left(l\right)=\overset{m}{\underset{i=0}{\sum }}\frac{l+ir}{l}{\left(p_{N}\right)}^{i}$$

(25) $$Q^{R}\left(l\right)=\frac{N}{p_{N}}\cdot \overset{m+1}{\underset{i=1}{\sum }}\frac{l+ir}{l}{\left(p_{N}\right)}^{i}$$

(26) $$Q^{C}\left(l\right)=Q^{S}\left(l\right)+Q^{R}\left(l\right)\left(1+\frac{1}{p}-\frac{p_{N}}{Np^{2}}\right)$$

(27) $$Q^{A}\left(l\right)=\frac{1}{p}\overset{m+1}{\underset{i=1}{\sum }}\frac{l+ir}{l}{\left(p_{N}\right)}^{i}$$

*where* $p_{N}=1-{\left(1-p\right)}^{N}$ *indicates the reliability of one-hop transmission.*

**Proof.**  Recursively expand the Equation (19):

$$Q^{S}\left(l+r\right)\;=\;\frac{l+2r}{l+r}\cdot \left(1-{\left(1-p\right)}^{N}\right)\cdot Q^{S}\left(l+2r\right)+1\;Q^{S}\left(l+2r\right)=\;\frac{l+3r}{l+2r}\cdot \left(1-{\left(1-p\right)}^{N}\right)\cdot Q^{S}\left(l+3r\right)+1\;\ldots \ldots \;Q^{S}\left(l+mr\right)=1\;\left(m=\lfloor \frac{R-l}{r}\rfloor \right)$$

To be brought in and removed in turn, the expression can be simplified as:

$$∴Q^{S}\left(l\right)=\overset{m}{\underset{i=0}{\sum }}\frac{l+ir}{l}{\left(1-{\left(1-p\right)}^{N}\right)}^{i}$$

Plugging $Q^{S}\left(l\right)$ into the Equation (20):

$$Q^{R}\left(l\right)=\;\frac{l+r}{l}\cdot N\cdot \overset{m}{\underset{i=0}{\sum }}\frac{l+\left(i+1\right)r}{l+r}{\left(1-{\left(1-p\right)}^{N}\right)}^{i}$$

$$Q^{R}\left(l\right)=\;\frac{N}{1-{\left(1-p\right)}^{N}}\cdot \overset{m}{\underset{i=0}{\sum }}\frac{l+\left(i+1\right)r}{l}{\left(1-{\left(1-p\right)}^{N}\right)}^{i+1}$$

$$Q^{R}\left(l\right)=\;\frac{N}{1-{\left(1-p\right)}^{N}}\cdot \overset{m+1}{\underset{i=1}{\sum }}\frac{l+r}{l}{\left(1-{\left(1-p\right)}^{N}\right)}^{i}$$

Putting tighter with Equation (21):

$$Q^{A}\left(l\right)=\frac{1-{\left(1-p\right)}^{N}}{Np}\frac{N}{1-{\left(1-p\right)}^{N}}\overset{m+1}{\underset{i=1}{\sum }}\frac{l+r}{l}{\left(1-{\left(1-p\right)}^{N}\right)}^{i}$$

$$Q^{A}\left(l\right)=\frac{1}{p}\overset{m+1}{\underset{i=1}{\sum }}\frac{l+ir}{l}{\left(1-{\left(1-p\right)}^{N}\right)}^{i}$$

□

**Figure 11:**
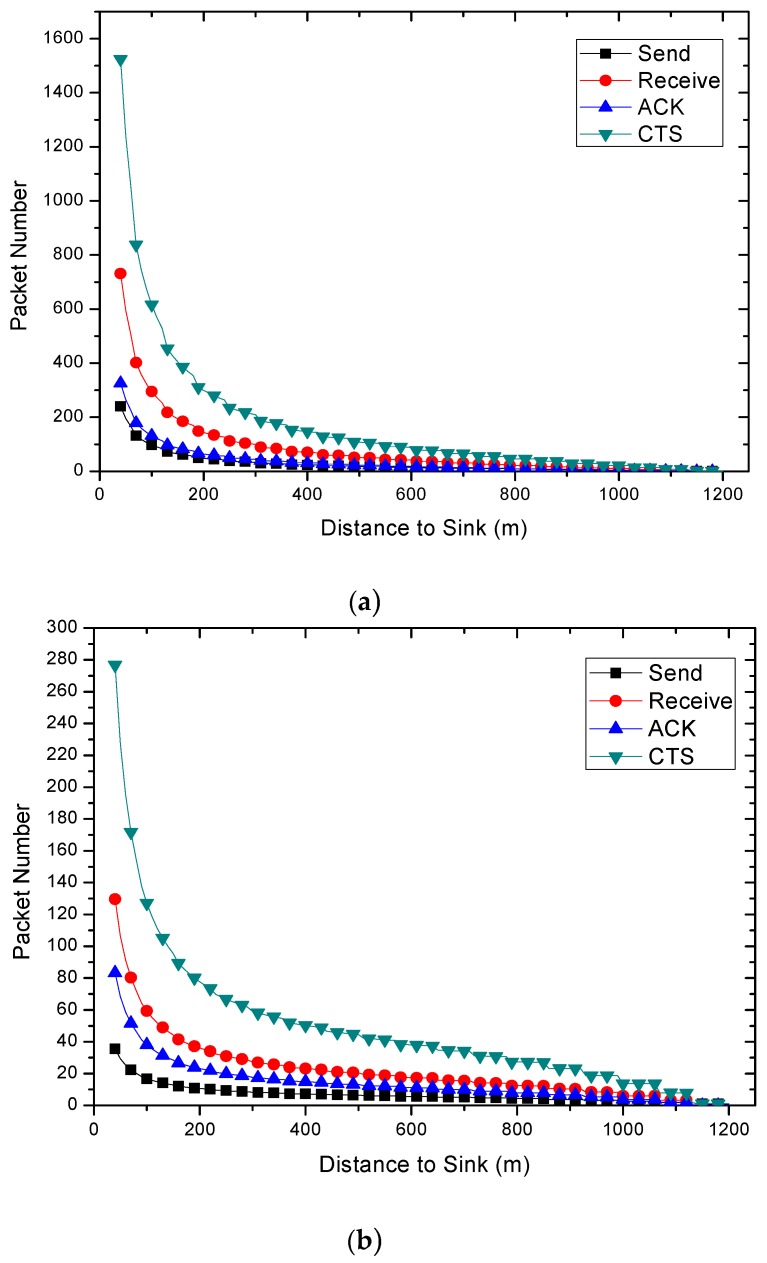
(**a**) Data volume of node under *N* = 3, *r* = 60; (**b**) Data volume of node under *N* = 3, *r* = 70.

Therefore, the data volume of the nodes using traditional routing method at different distances from the sink in a round of sending can be calculated. From Figure 6 and Figure 11, the influence of transmission distance and size of candidate set on packet load is generated. The shorter the transmission distance and the more candidate nodes make the wireless network transmission more reliable. As the data loss rate becomes smaller, the amount of data on the network also becomes larger.

**Figure 12:**
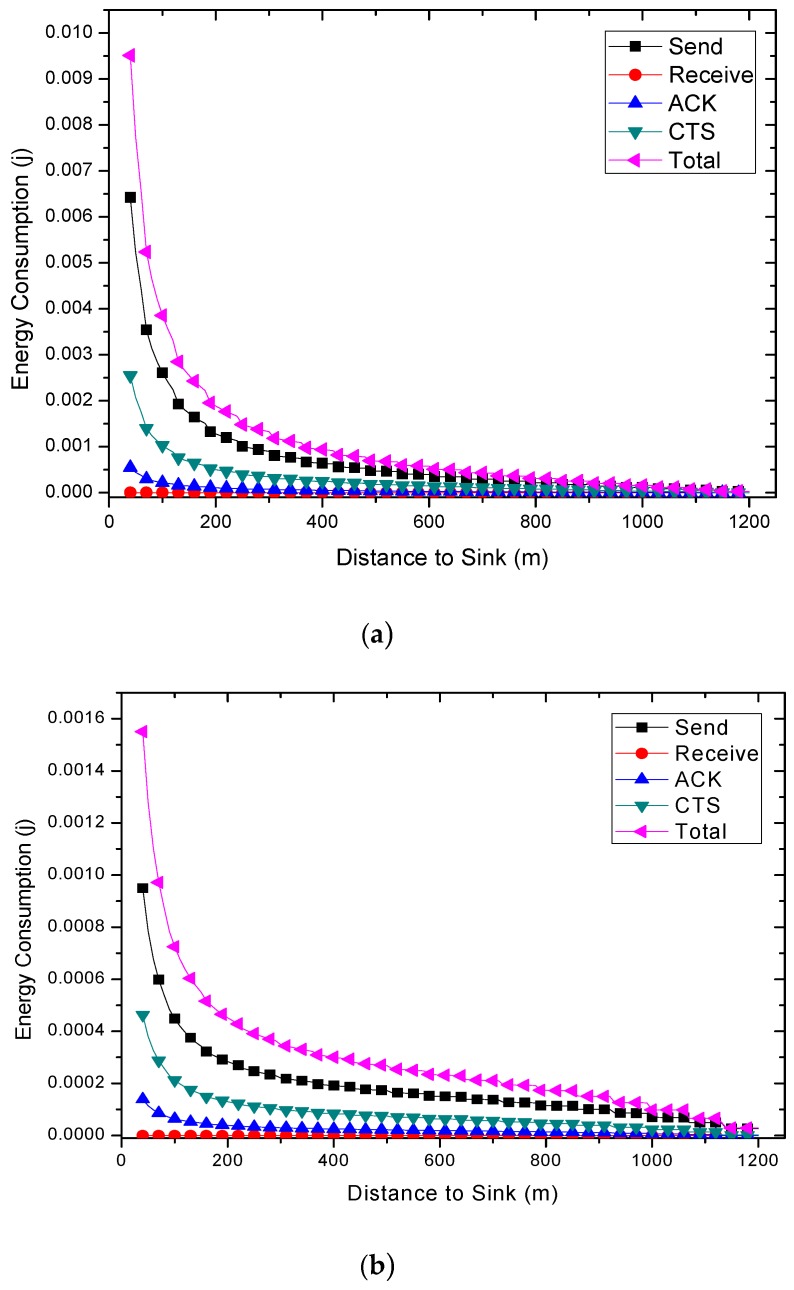
(**a**) Energy consumption of node under *N* = 3, *r* = 60; (**b**) Energy consumption of node under *N* = 3, *r* = 70.

Combined with Equations (6)–(10), the energy consumption of nodes at different distances from sink can be calculated. The results are shown in Figure 7 and Figure 12. Due to different network parameters, the level of energy consumption of single round may be different, so it is not intuitive enough to use the subtraction value to measure the residual energy. In this paper, the energy residual degree $\lambda_{l}$ is adopted to measure the residual energy of the node $\;n_{l}$.

**Definition** **1.** *The energyresidualdegree*$\lambda_{l}$*of the node*$n_{l}$*is defined as the ratio of the energy saved by the node*$n_{l}$*compared to the maximum energy consumption to the energy* consumed *by the node itself in single sending round. Generally, the value of* $\lambda_{l}$ *is computed as:*

(28) $$\lambda_{l}=\frac{\max\left(E_{l}^{TOT}\right)-E_{l}^{TOT}}{E_{l}^{TOT}}$$

**Figure 13:**
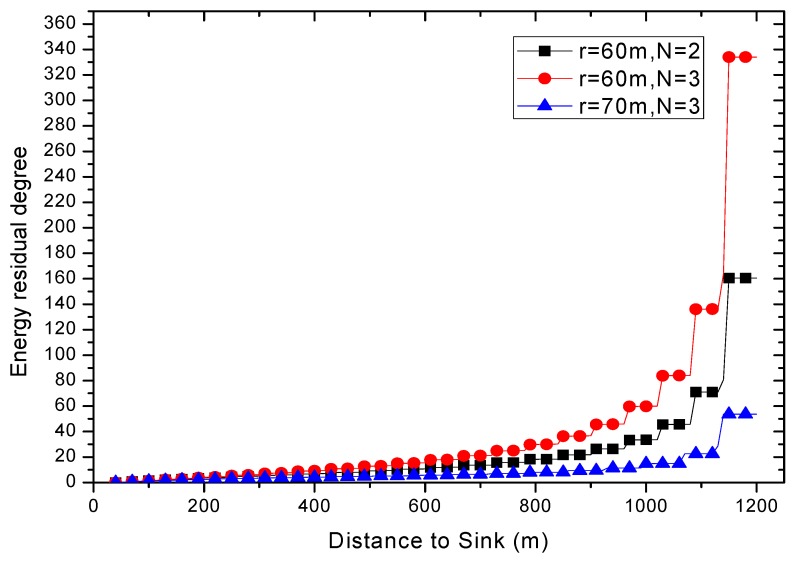
Energy residual degree of node at different distance.

It can be seen from the Figure 13 that the residual energy of the network edge nodes will increase dramatically, but the energy consumption of the nodes in the near sink region is relatively small. Therefore, the reliability of the transmission can be improved by increasing the transmission power of the node to different degrees, and the farther the node is from the sink, the higher the transmission power is.

The core part of the COOR scheme is to calculate the transmission power of nodes at different distances. Considering the COOR(R) method, since the receiving and forwarding structure of the network is mainly determined by the transmission radius, as long as it remains unchanged, the forwarding region assumed by each region in a round of transmission will not change. However, when the transmission power of the nodes is increased, the reliability of the network is improved. As the amount of data in the network increases, the energy consumption of the nodes is bound to raise. In view of this situation, when calculating the transmission power, it is necessary to estimate the amount of data in the optimized network and select the reference threshold energy.

**Theorem** **4.** *Suppose the value of reference threshold energy is* $E_{\theta }^{TOT}$ *. In COOR (R) method, the transmit power of a node* $n_{l}$ *with a distance* l *to the sink is:*

(29) $$P_{t}{\left(l\right)}_{w}=\frac{R_{D}\;\cdot \;E_{\theta }^{TOT}+PL\left(r\right)\cdot L^{r}Q_{l}^{R}}{L^{s}Q_{l}^{S}+L^{r}\cdot Q_{l}^{R}+L^{CTS}Q_{l}^{C}+L^{ACK}Q_{l}^{A}}$$

*where* $Q_{l}^{S},\;Q_{l}^{R},\;Q_{l}^{C},\;Q_{l}^{A}$ *respectively indicates the number of send, receive, CTS, ACK packets estimated for the node* $n_{l}$ *.*

**Proof.**  Putting Equations (6)–(10) together, the estimated energy consumption of the node $n_{l}$ under unsteady power can be expressed as:

$$E_{l}^{TOT}=\;E_{l}^{s}+E_{l}^{r}+E_{l}^{CTS}+E_{l}^{ACK}$$

$$E_{l}^{s}+E_{l}^{CTS}+E_{l}^{ACK}=\;\frac{P_{t}\left(l\right)\cdot \left(L^{s}Q_{l}^{S}+L^{CTS}Q_{l}^{C}+L^{ACK}Q_{l}^{A}\right)}{R_{D}}$$

$$E_{l}^{r}=\frac{P_{r}\left(l\right)\cdot L^{r}Q_{l}^{R}}{R_{D}}\;=\frac{(P_{t}\left(l\right)-PL\left(r\right))\cdot L^{r}Q_{l}^{R}}{R_{D}}$$

$$E_{l}^{TOT}=\;\frac{P_{t}\left(l\right)\cdot \left(L^{s}Q_{l}^{S}+L^{r}Q_{l}^{R}+L^{CTS}Q_{l}^{C}+L^{ACK}Q_{l}^{A}\right)-PL\left(r\right)L^{r}Q_{l}^{R}}{R_{D}}$$

When $P_{t}\left(l\right)$ equals $\frac{R_{D}\;\cdot \;E_{\theta }^{TOT}+PL\left(r\right)\cdot L^{r}Q_{l}^{R}}{L^{s}Q_{l}^{S}+L^{r}\cdot Q_{l}^{R}+L^{CTS}Q_{l}^{C}+L^{ACK}Q_{l}^{A}}$, if the estimated amount of data is completely accurate, then the energy consumptions of all the nodes will be exactly balanced (values $E_{\theta }^{TOT}$). However, the data volume of the node is influenced by the transmission success rate, and the transmission success rate is directly related to the transmit power. Therefore, a heuristic method for estimating data volume is provided to obtain an approximate optimal solution of transmission power. □

According to Theorem 2, to estimate the data volume of the node one only needs to evaluate the reliability of transmission. Obviously, the transmission success rate of nodes near the sink is basically unchanged, and it reaches the upper limit 1 as the distance gradually increases.

Therefore, the binary search method can be used to quickly enumerate the distance l, at which the reliability of transmission reaches 1. Assuming that the transmission success rate changes linearly and remains at 1 after l, then the predicted value and the calculated value can be obtained. Comparing the two values, the estimation is unacceptable unless the error is less than 3%. By this way, all nodes’ transmit power are determined.

As mentioned above, the improvement of the transmit power of the node increases with its distance to the sink, and the transmission distance of the node is fixed. Therefore, the reliability of transmission will increase with the distance to the sink. At the same time, according to the energy formula, if the amount of data carried by the node is overestimated, the node will have an energy surplus.

The reference threshold energy can select the value of maximum energy consumption in the traditional routing method, that is $E_{\theta }^{TOT}=\max\left(E_{l}^{TOT}\right)$. When the reliability of the network is relatively low, the reference energy should be properly increased.

**Figure 14:**
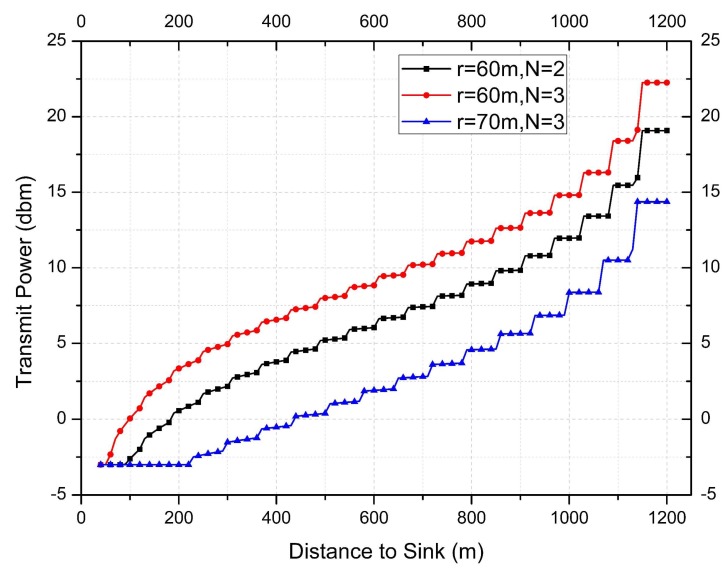
Transmit power of node at different distance in COOR(R) method.

**Figure 15:**
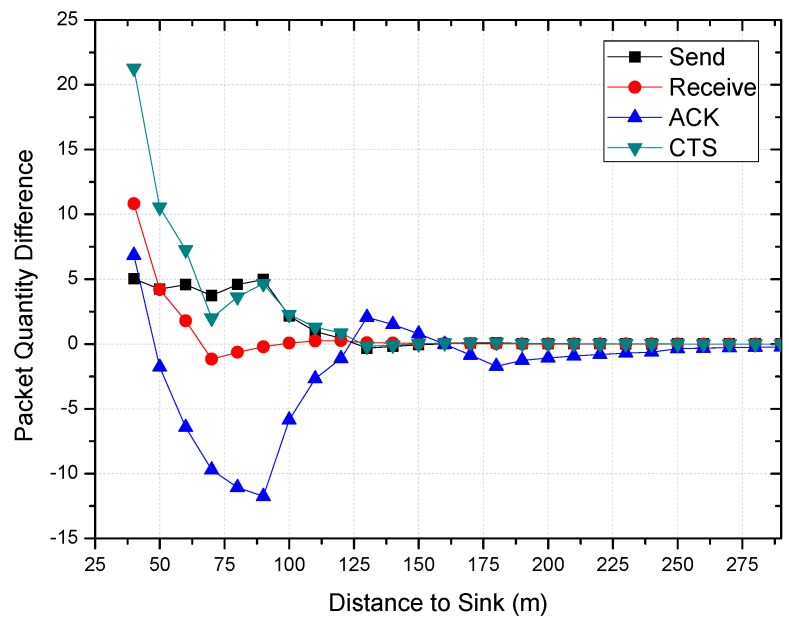
Packet quantity difference under *N* = 2, *r* = 60 in COOR(R) method.

Figure 14 indicates the transmission power under the COOR (R) method when the initial value of the nearest sink node is $P_{t}=-3\;\mathrm{dBm}$. The result of packet quantity difference (predicted value—calculated value) is given in Figure 15. In the COOR (P) method, since the transmission distance is increased, the original network structure is changed.

**Theorem** **5.** *Considering the transmit power of the traditional routing method is* $P_{t}{}_{dbm}$ *, and the node* $n_{l}$ *is reset to* $P_{t}{\left(l\right)}_{dbm}$ *after updated by COOR(P) method. Then the transmission radius of node* $n_{l}$ *can be obtained:*

(30) $$x_{l}=r\cdot 10^{\frac{1}{10n}(P_{t}\left(l\right)-P_{t})}$$

**Proof.**  According to Equation (4), maintaining the PAR means the SNR of the communication link remains unchanged. Putting Equations (1) and (2) together:

$$P_{t}\left(l\right)-PL{\left(x_{l}\right)}_{dB}-P_{n}{}_{dB}=P_{t}-PL{\left(r\right)}_{dB}-P_{n}{}_{dB}$$

$$P_{t}\left(l\right)-10n\cdot \log_{10}\left(\frac{x_{l}}{d_{0}}\right)=P_{t}-10n\cdot \log_{10}\left(\frac{r}{d_{0}}\right)$$

$$\frac{1}{10n}(P_{t}\left(l\right)-P_{t})=\log_{10}\frac{x_{l}}{r}$$

$$x_{l}=r\cdot 10^{\frac{1}{10n}(P_{t}\left(l\right)-P_{t})}$$

□

In the COOR(P) method, the receiving and forwarding structure of the network has changed, it is necessary to assume that the regions to be forwarded by the area $A_{l,k}$ are $A_{l+u,k}$, then the data volume of node $n_{l}$ can be calculated as follows:

(31) $$Q^{S}\left(l\right)=1+\left(1-{\left(1-p\right)}^{N}\right)\sum \frac{l+u}{l}\cdot Q^{S}\left(l+u\right)$$

(32) $$Q^{R}\left(l\right)=N\sum \frac{l+u}{l}\cdot Q^{S}\left(l+u\right)$$

(33) $$Q^{C}\left(l\right)=Q^{S}\left(l\right)+Q^{R}\left(l\right)\left(1+\frac{1}{p}-\frac{1-{\left(1-p\right)}^{N}}{Np^{2}}\right)$$

(34) $$Q^{A}\left(l\right)=Q^{R}\left(l\right)\cdot \frac{1-{\left(1-p\right)}^{N}}{Np}$$

**Figure 16:**
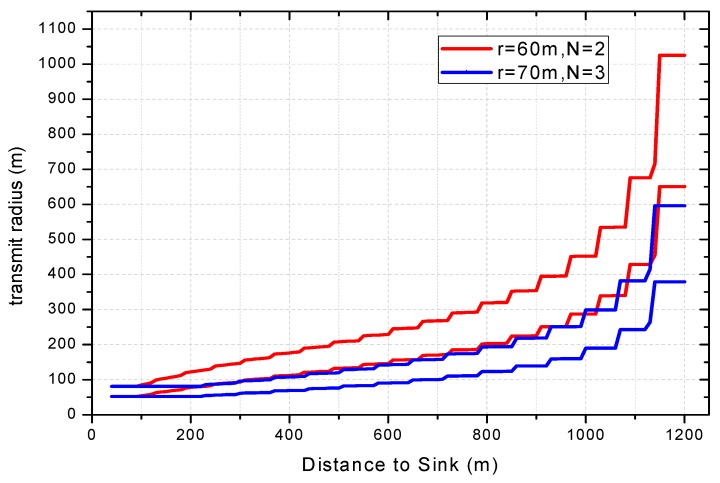
Transmission radius of node at different distance in COOR(R) method.

Similar to Theorem 4, the heuristic method is adopted to find the approximate optimal transmit power $P_{t}{\left(l\right)}_{w}$ of each node in the COOR(P) method. The change of node transition area under COOR(P) method is shown in Figure 16. In general, the farther a node is from the sink, the larger its transition area is.

### 5.2. COOR Protocol

The main goal of the COOR scheme is to make full use of the unbalanced load of the network, and to improve the network performance by changing the transmission power of nodes in different areas of the network. Based on the different emphasis on network performance, two optimization methods are provided. Specifically, the implementation of the two methods is given by Algorithm 1.

The priority of candidate nodes is not fixed, mainly determined by the effective forwarding distance and the current energy surplus.

**Definition** **2.** *Suppose that* d(S,sink) *represents the distance from node* S *to the sink,* $d_{i}$ *represents the effective forwarding distance of node* $n_{l}$ *selecting candidate node* $C_{i}$ *, and* $\psi_{i}$ *represents the current remaining energy of node* $C_{i}$ *. The priority of the node* $C_{i}$ *under the COOR scheme is:*

(35) $$Pri_{i}=\alpha \cdot d_{i}+\beta \cdot \psi_{i}$$

*where the effective forwarding distance is:*

(36) $$d_{i}=d\left(n_{l},\;sink\right)-d\left(C_{i},sink\right)$$

*Here,* α *and* β *are weight factors, they can adjust the importance of effective forwarding distance and residual energy in the priority calculation. Meanwhile, it can be known from the formula that the longer the forwarding distance and the more residual energy, the higher the priority.*

In order to maintain the reliable communication between candidate nodes, the distance between candidate nodes need to be limited to distance $D_{rd}$. That is $d\left(C_{i},C_{j}\right)<D_{rd}$. In the COOR protocol, the transmit power of each node is different, thus the size of the communication area is different.

However, there is no significant difference in transmit power between the candidate nodes, and the communication message is much smaller than the data packet. Therefore, a reasonable approach is to select the maximum transmission radius of the sending node $r_{max}$ as the limit distance $D_{rd}$ between the forwarding nodes.

**Algorithm** **1:** COOR scheme for network communication.
1:Using COOR (R) method or COOR (P) method to calculate the power of the node, set the optimal transmit power.2:**If** COOR(P) method is adopted **then**3:  Adjust the sending radius of the node;4:
**End if**
5:Sensors start monitoring, and the wireless sensor network is regard alive until one of the sensor nodes run out of its energy.6:**While** The energy of the node is not exhausted7: The sending node generates a packet according to specific application;8: **For** each data transmission **Do:**9:  **If** Data fusion required **then**10:    Reorganizes the current data packet with local stored information;11:  **End if**12:  Reads the local information of forwarding distance and the residual energy;13:  Calculates the priority of each node in the transition region;14:  Selects the highest priority N nodes to form the candidate node set;15:  Sender broadcasts Request to Send (RTS) message to all candidate nodes and wait;16:  Receiver replies a Clear to Send (CTS) message to the Sender when it is free, where the updated information of residual energy is attached;17:  After receiving all the CTS messages, Sender broadcast data packets to all candidate nodes. Here, the priority information of each candidate node is appended to the data packet;18:  After data reception is completed, candidate nodes broadcast its result in order of priority.19:  **For** each candidate nodes **Do:**20:   **If** the reception succeeds **then**21:    Broadcast the ACK message to all candidate nodes and the send node. The remaining candidate nodes delete the duplicate data packets;22:   **Else**23:    Check its priority;24:    **If** is the lowest **then**25:     Broadcast the final NACK message to the send node;26:    **Else**27:     Broadcast the NACK message to remaining candidate nodes. The next priority node starts checking its own reception;28:    **End if**29:    The sending node records the results of the current data transmission;30:   **End if**31:  **Until** the ACK message or final NACK message is replied; **//** For each candidate nodes32: **Until** the current data packet reaching the sink or lost; // For each data transmission33:**End** **while** **//** The energy of the node is not exhausted


## 6. Performance Analysis and Comparison

The following in this chapter is a comprehensive comparison and analysis of the performance of COOR and previous strategies, including reliability comparison, delay comparison and energy consumption analysis. The main notations adopted in simulation are concluded in Table 2. If no additional explanation is given, the default value of experimental parameters are referred in Table 2.

In addition, when comparing the partitioning performance, the network is divided into 6 tiers, that is, the width of each partition is 200 m. Due to the symmetrical structure, consider the node’s line density ρ as one node per 10 m. At the same times, the experiment simulations are performed on the basis of MATLAB software.

### 6.1. Network Weighted Average Performance

Before presenting the results of the experiment, this section firstly introduces the calculation method of network partition reliability and delay. The reception rate of data packets and the hop count are respectively used as the measure of reliability and delay. In addition, partitions are mainly based on the distance to the sink as well as the physical location of the nodes in the network. As shown in Figure 9, in the calculation of partition performance, the circular network area is divided into multiple rings with equal width, and each ring is regard as a partition. In general, this paper uses $pat_{x}^{y}$ to represent a partition whose distance to the sink is between x m and y m.

**Definition** **3.** *When measuring partition performance, the weight of each node is equal. Consider that the performance of node* $n_{l}$ *whose distance to Sink is* $Fet^{l}$ *, the overall performance* $Fet_{x}^{y}$ *of partition* $pat_{x}^{y}$ *is calculated as:*

(37) $$Fet_{x}^{y}=\frac{\int_{x}^{y}\int_{0}^{2\pi }Fet^{l}\cdot \rho l\cdot dl\cdot d\theta }{\rho \pi y^{2}-\rho \pi x^{2}}$$

**Theorem** **6.** *Consider that $\psi \left(l,P_{t},r\right)$* *denotes the reliability of the node whose distance to the sink is* l *, the transmission radius is* r *, and the transmission power is* $P_{t}$ *. Then, the overall reliability* $\psi_{x}^{y}$ *of the* *partition* $pat_{x}^{y}$ *calculated in a discrete form can be expressed as:*

(38) $$\psi_{x}^{y}=\frac{2\;\sum_{i=x}^{y}\psi \left(i,P_{t},r\right)\cdot i}{\left(y+x\right)\cdot \left(y-x+1\right)}$$

**Proof.**  Putting Equations (4), (5) and (35) together, the reliability of partition is obtained:

$$\psi_{x}^{y}=\frac{\int_{x}^{y}\int_{0}^{2\pi }\psi \left(l,P_{t},r\right)\rho l\cdot dl\cdot d_{\theta }}{\rho \pi y^{2}-\rho \pi x^{2}}$$

Due to the discontinuity of $\psi \left(l,P_{t},r\right)$ and the actual distribution of nodes, it is necessary to perform discretization when calculating. At the same time, it should be noted that during the conversion process, deviations may occur, which may result in a reliability value exceeding 1.

$$\mathrm{Assume}\;\forall i\;\psi \left(i,P_{t},r\right)=1\;\frac{2\pi \sum_{i=0}^{R}\psi \left(i,P_{t},r\right)\cdot \rho \cdot i}{\rho \pi y^{2}-\rho \pi x^{2}}\;=\;\frac{2\;\sum_{i=0}^{R}i}{y^{2}-x^{2}}=\;\frac{y-x+1}{y-x}>1$$

After eliminating the deviation of conversion:

$$\psi_{x}^{y}=\frac{2\pi \sum_{i=x}^{y}\psi \left(i,P_{t},r\right)\cdot \rho i}{\rho \pi y^{2}-\rho \pi x^{2}}\times \frac{y-x}{y-x+1}$$

$$\psi_{x}^{y}=\frac{2\sum_{i=x}^{y}\psi \left(i,P_{t},r\right)\cdot i}{\left(y+x\right)\cdot \left(y-x\right)}\times \frac{y-x}{y-x+1}\;$$

$$\psi_{x}^{y}=\frac{2\;\sum_{i=x}^{y}\psi \left(i,P_{t},r\right)\cdot i}{\left(y+x\right)\cdot \left(y-x+1\right)}$$

□

Similarly, the overall reliability $D_{x}^{y}$ of the partition $pat_{x}^{y}$ calculated in a discrete form can be expressed as:

(39) $$D_{x}^{y}=\frac{2\;\sum_{i=x}^{y}D\left(i,P_{t},r\right)\cdot i}{\left(y+x\right)\cdot \left(y-x+1\right)}$$

Here, $D\left(l,P_{t},r\right)$ indicates the delay of the node whose distance to the sink is l, the transmission radius is r, and the transmission power is $P_{t}$.

### 6.2. End-to-End Reliability

After the COOR scheme is applied, the *PAR*, one-hop reliability, and the end-to-end reliability have been significantly improved especially in far-sink region. The end-to-end reliability of node and partition are compared separately.

**Figure 17:**
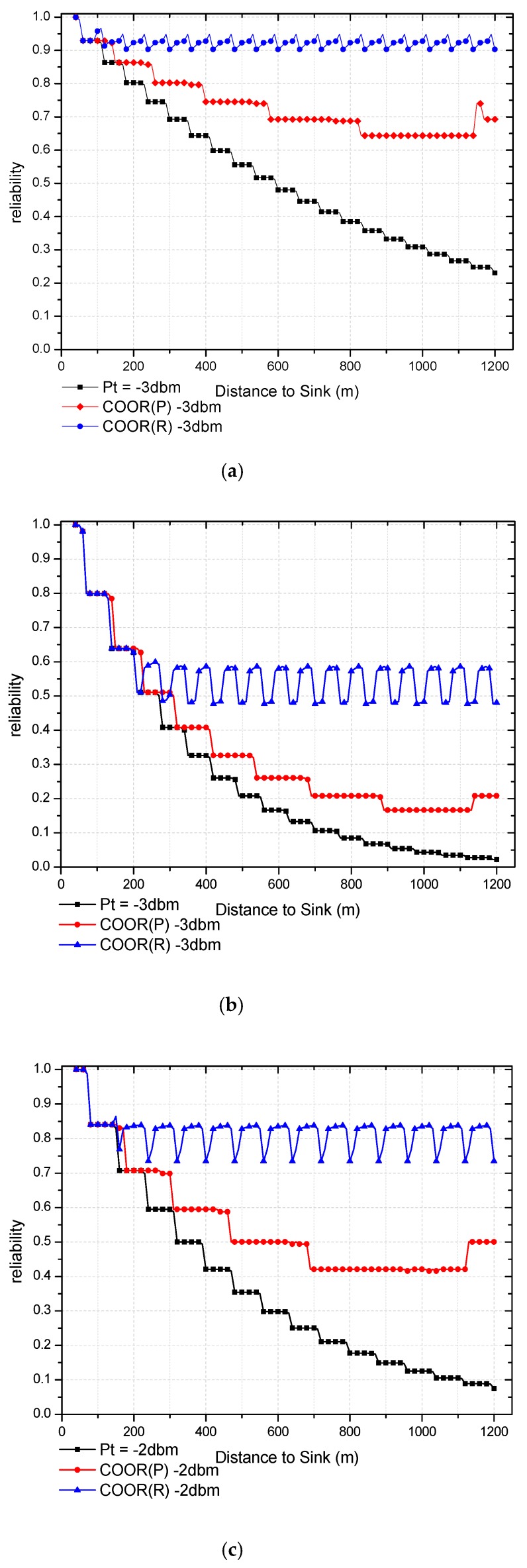

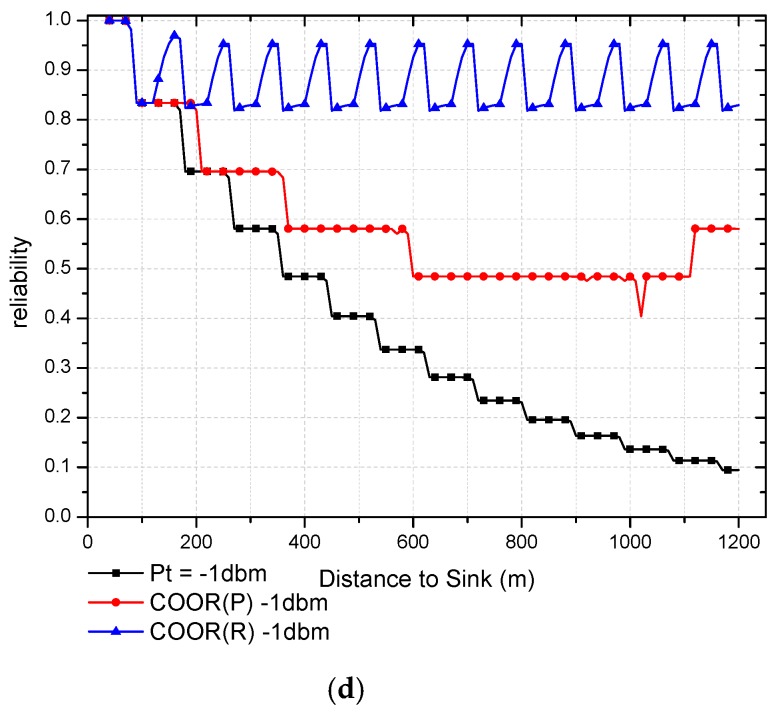
(**a**) End-to-end reliability of node at different distance under *r* = 60, *N* = 2; (**b**) End-to-end reliability of node at different distance under *r* = 70, *N* = 3; (**c**) End-to-end reliability of node at different distance under *r* = 80, *N* = 4; (**d**) End-to-end reliability of node at different distance under *r* = 90, *N* = 4.

As can be seen from Figure 17, both COOR(R) method and COOR(P) method have an improvement on end-to-end reliability, which means the data collection rate is increased.

When the network itself has certain reliability, the performance of COOR method is significant. As shown in Figure 17a, when the distance to the sink is 200 m, the end-to-end reliabilities of the traditional routing method, COOR(P) method, and COOR(R) method were 0.802, 0.863, 0.923, respectively, which were 7.62% and 15.07% of the increase rate. As the distance to the sink reaches 1000 m, the end-to-end reliability gradually reaches 0.308, while the COOR(P) method and COOR(R) method maintain values of 0.643 and 0.927, which renders a 108.05% and 200.04% increase rate.

Compared with Figure 17a, the information of improvement on low-reliability network can be obtained in Figure 17b. Due to the low reliability of the previous network, the selected threshold reference energy is low, which limits the improvement of the network by the COOR strategy. It can be seen that the COOR scheme begins to gradually improve the network reliability until the distance to sink reaching 240 m. However, with a distance of 900 m to the sink, the end-to-end reliability of traditional routing method, COOR(P) method, COOR(R) method were respectively 0.066, 0.166, 0.581, which represent a 149.78% and 774.41% increase rate. This is mainly due to the rapid decline in the reliability of the previous unreliable network and the fact the COOR scheme mainly improves the performance of the far-sink region nodes.

Obviously, in the COOR(R) method, the transmission success rate is significantly improved by the increased transmission power. When the transmission power exceeds a certain value, the reliability of the single hop link will reach 100%. At this point the end-to-end reliability of the current node depends on the end-to-end reliability of the next hop node.

Therefore, the end-to-end reliability of the nodes in far-sink area appears periodic, and it will rise at the edge distance (k·r). Since the reliability ($\psi_{k\cdot r+\varepsilon }$) of the node at k·r+ε is equal to the reliability ($\psi_{\left(k-1\right)\cdot r+\varepsilon }$) of the node at (k−1)·r+ε multiplied by the reliability of the first hop of the current node ($\zeta_{1}$). That is, when $\zeta_{1}>\psi_{\left(k-1\right)\cdot r+\varepsilon }/\psi_{k\cdot r-\varepsilon }$, the reliability of the node is higher than the near one, but the reliability of the nodes in each cycle will be strictly not greater than the reliability of the nodes in the previous cycle.

**Figure 18:**
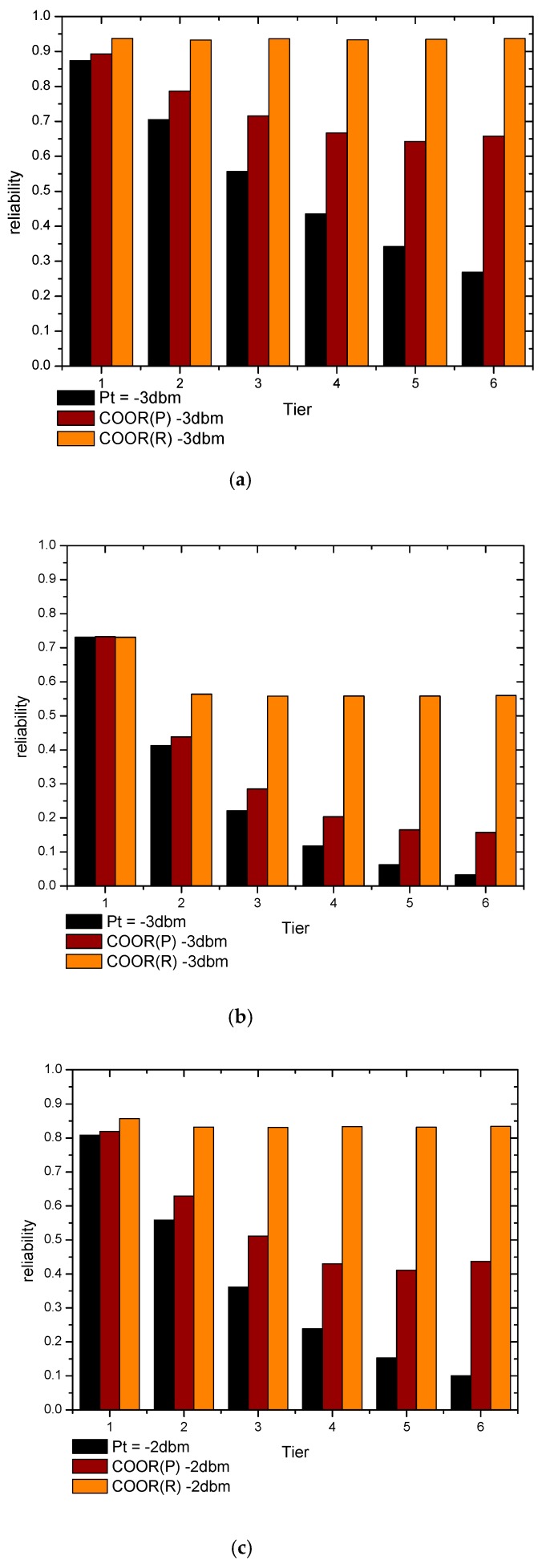

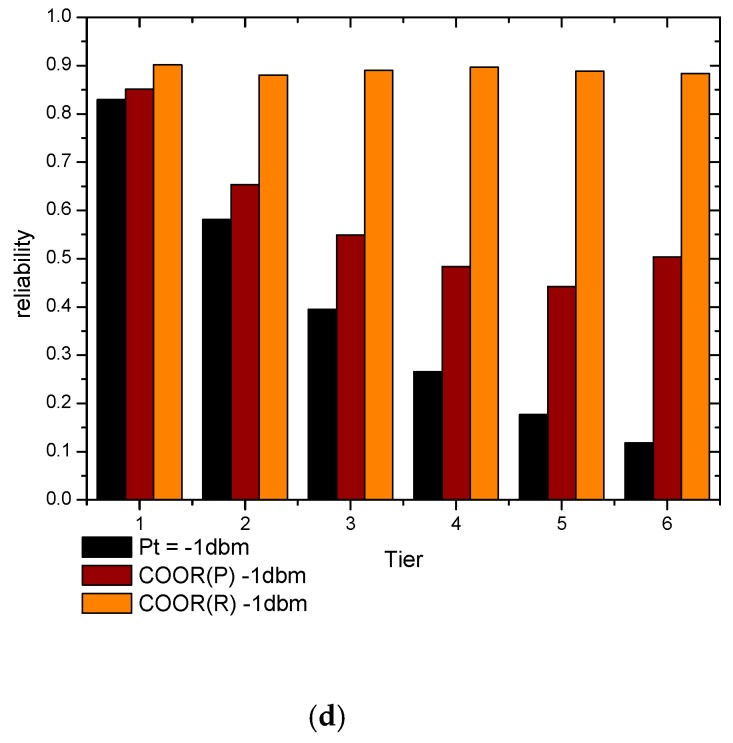
(**a**) End-to-end reliability of different region under *r* = 60, *N* = 2; (**b**) End-to-end reliability of different region under *r* = 70, *N* = 3; (**c**) End-to-end reliability of different region under *r* = 80, *N* = 4; (**d**) End-to-end reliability of different region under *r* = 90, *N* = 4.

On the other hand, the COOR(P) method keeps the transmission success rate unchanged and improves the reliability by reducing the number of hops. Consequently, in the near-sink region, the increase of the transmission distance is small, and the improvement of reliability is not obvious. With the gradual increase of the transmission power, the reliability also becomes more and more obvious. Meanwhile, the reliability of the previous network has a greater impact on this method. In Figure 18, the changes in the reliability of different region in the network are shown.

As can be seen from Figure 18a, the end-to-end reliabilities in tier1 (T1) are almost same. In the COOR(P) method, the end-to-end reliability of nodes in T1 increased from 0.874 to 0.893, which is 2.23% higher. As for the COOR(R) method, it maintained a reliability value of 0.937 with a 7.31% increase rate. When it comes to the far-sink region (T5), the end-to-end reliabilities of the traditional routing method, COOR(P) method, COOR(R) method were respectively 0.342, 0.643, 0.934, which represent a 87.77% and 173.07% increase rate.

From a regional perspective, it can be seen from Figure 18b that the COOR method increases the reliability of the network from outside to inside. The reliability of T1 can be regard as the same, while in T2, the traditional routing method, COOR(P) method, COOR(R) method respectively maintained the end-to-end reliability at values of 0.413, 0.438, 0.564, which were a 6.18% and 36.62% increase rate. Furthermore, when the COOR(P) method provided a value of 0.165 in T5, the reliability in the COOR(R) method was still kept at 0.558, while in the traditional method it decreased to 0.062.

In general, the improvement of the reliability of the outer nodes requires the consumption of the remaining energy in all inner areas, especially when the network is not reliable under the traditional routing method. Specifically, due to the significant increase in the number of data packets brought about by the increase in reliability, the transmission power of the nodes in the near-sink region cannot be increased, so the reliability remains unchanged. As the distance to the sink increases further, the remaining energy is sufficient enough to further improve the performance of reliability. From the perspective of data collection rate, the COOR scheme mainly improves the reception rate of data packets sent by nodes in far-sink region of the network, which are easily lost under traditional routing method.

### 6.3. Transmission Delay

Since the hop count is adopted as a measure of delay in this paper, the delay of the node does not change without changing the network structure, which is determined by the transmission distance. That is, the COOR(R) method has an exactly the same delay as the traditional routing method.

**Figure 19:**
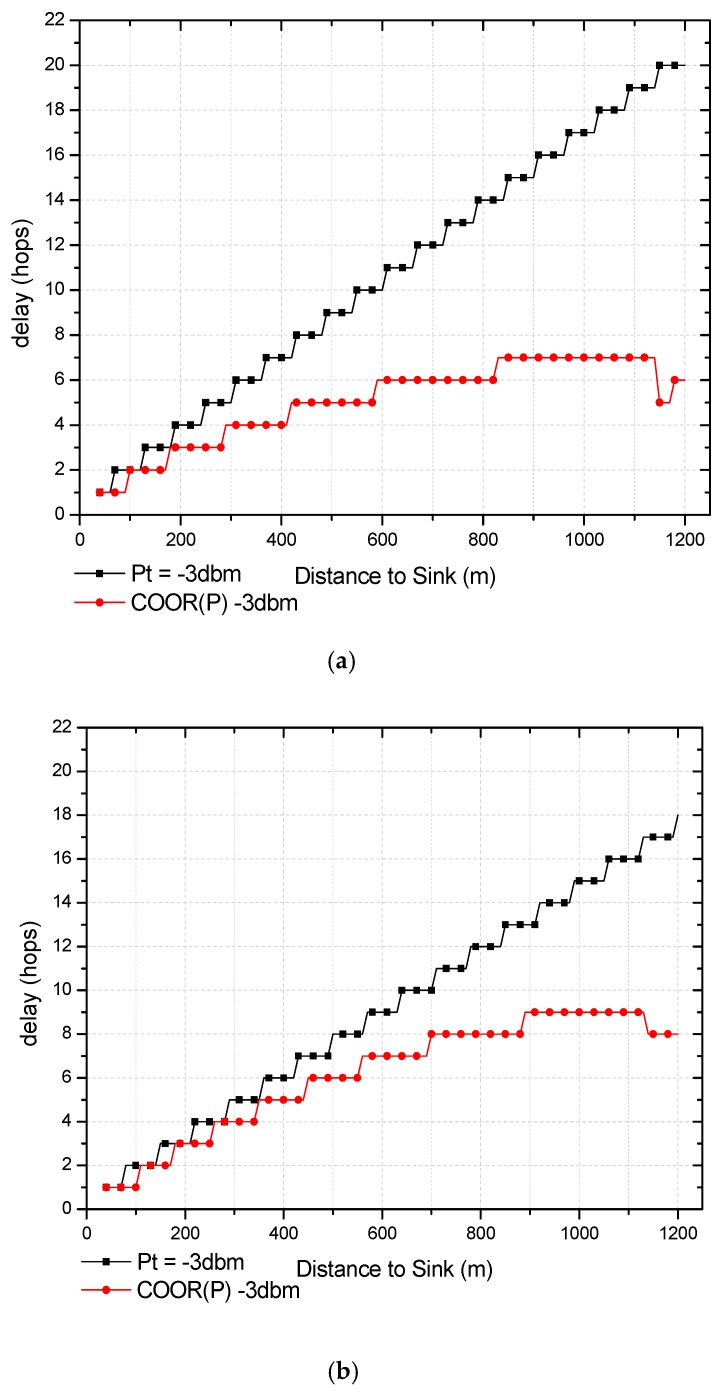

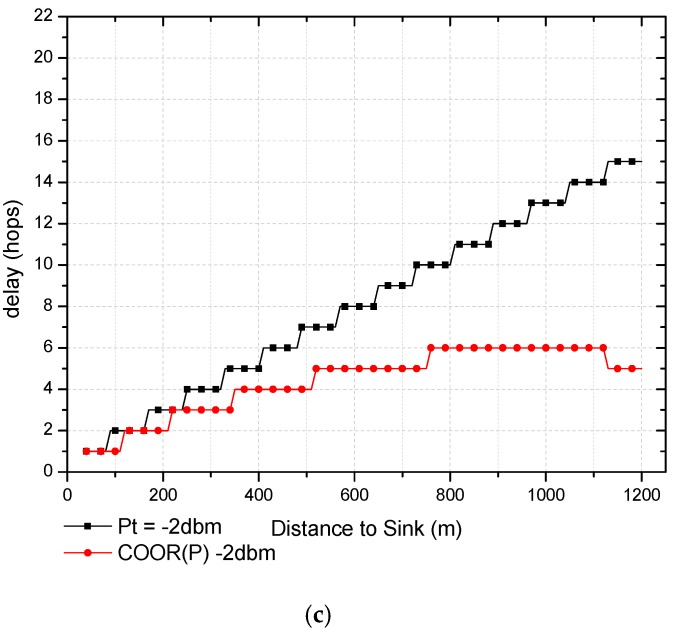

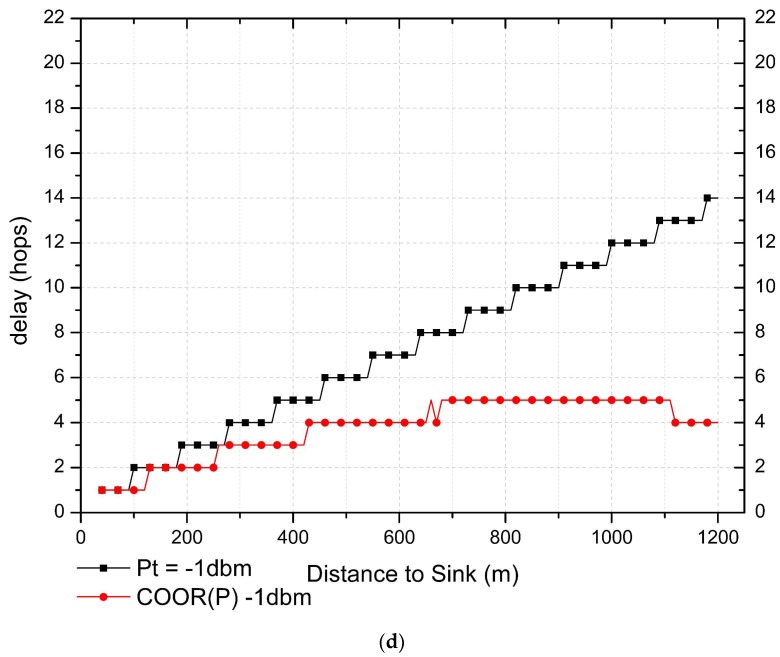
(**a**) Delay of node at different distance under *r* = 60, *N* = 2; (**b**) Delay of node at different distance under *r* = 70, *N* = 3; (**c**) Delay of node at different distance under *r* = 80, *N* = 4; (**d**) Delay of node at different distance under *r* = 90, *N* = 4.

In fact, however, the COOR(R) method reduces the time for each hop and therefore has a smaller improvement effect on network delay. This section mainly shows the delay performance comparison between the COOR(P) method and the previous routing method. In Figure 19, the changes of the end-to-end delay of nodes after using COOR(P) method are compared at different distances.

As shown in Figure 19a, the delay decreased from nine hops to five hops, and the decrease was 80% at 500 m to the sink. When the distance to the sink is 1000 m, the end-to-end delay of traditional routing method and COOR(P) method are respectively 17 hops and seven hops, which is 142% decrease rate. At the extreme edge of the network, the delay of the node may be lower than that of the near node, which results from the different levels of increase in transmission power.

Similarly, when optimizing the unreliable network, the COOR(P) method will also have a limited improvement effect on delay. According to Figure 19b, the end-to-end delay of traditional routing method and COOR(P) method were respectively seven hops and six hops, which was a 16.7% decrease rate with a distance of 500 m to the sink. Meanwhile, when the distance to the sink reached 1000 m, the delay decreased from nine hops to five hops, and the decrease was 80%. Compared to the situation with r=60 N=2, the performance improvement is much smaller.

As mentioned above, in the COOR(P) method, the farther the distance from the sink in the network is, the higher the transmission power of the node is. Meanwhile, because the transmission success rate is kept constant, the greater the transmission power, the farther the transmission distance will be. Consequently, the delay of the nodes in the far-sink region is greatly improved.

**Figure 20:**
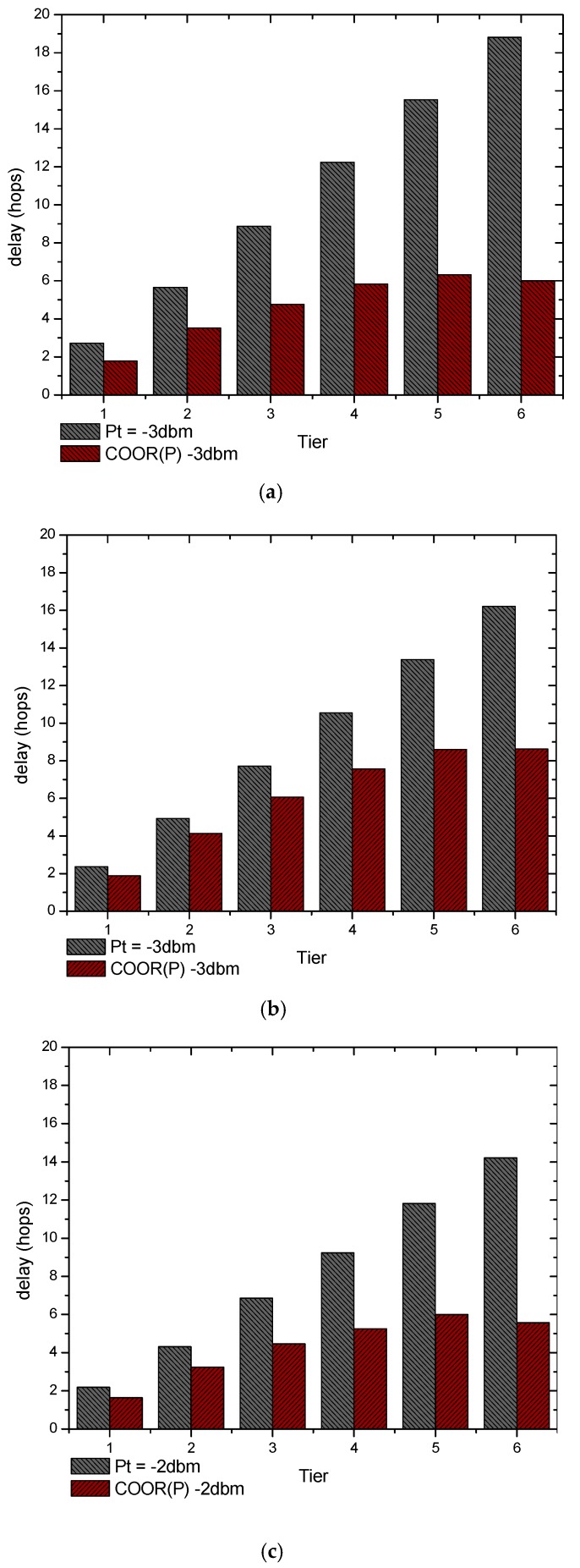

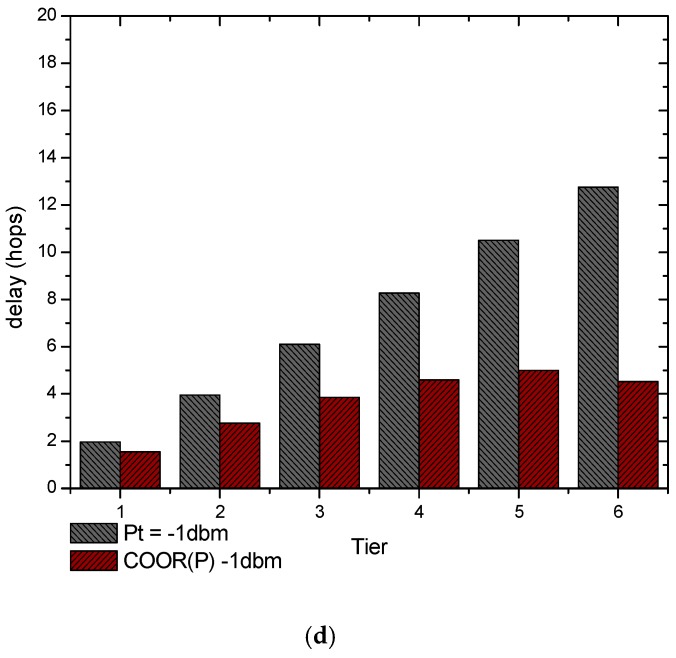
(**a**) Delay of different region under *r* = 60, *N* = 2; (**b**) Delay of different region under *r* = 70, *N* = 3; (**c**) Delay of different region under *r* = 80, *N* = 4; (**d**) Delay of different region under *r* = 90, *N* = 4.

Furthermore, the changes in the delay of different region in the network are shown in Figure 20.

As shown in Figure 20a, the delay in traditional method increased linearly, while under COOR(P) method the delay increased more slowly and were kept at a certain value in the far-sink region. The end-to-end delay of nodes in T1 decreased from 1.968 to 1.553, which is 21.09% lower. As for the far-sink region (T5), the delay of traditional routing method and COOR(P) method were respectively 10.512, 4.994, which represent a 52.48% reduction rate.

Similarly, the unreliable networks also limit the improvement of delay performance. In Figure 20b, the decrease rates are respectively 20.58%, 21.48%, and 35.68% in T1, T3 and T5. Compared to the circumstance in Figure 20a, the delay reduction in the traditional routing method naturally results from the longer transmission distance in each hop, while the delay increased (still lower than the traditional method) mainly due to the relatively small reference threshold energy. Specifically, when the transmission distance increased with the same transmission power, the reliability of communication links decreases, which renders a smaller data volume in the network. As a consequence, the small reference threshold energy limits the optimization of the COOR(P) method.

In conclusion, the delay improvements are related to the network reliability. As the distance to the sink increases, a fixed larger transmission distance can bring about a smaller delay. While in the COOR(P) method, the transmission distance even increases with the distance to the sink, the COOR(P) method significantly reduces the delay of the nodes in the far-sink region of the network.

### 6.4. Energy Consumption

**Figure 21:**
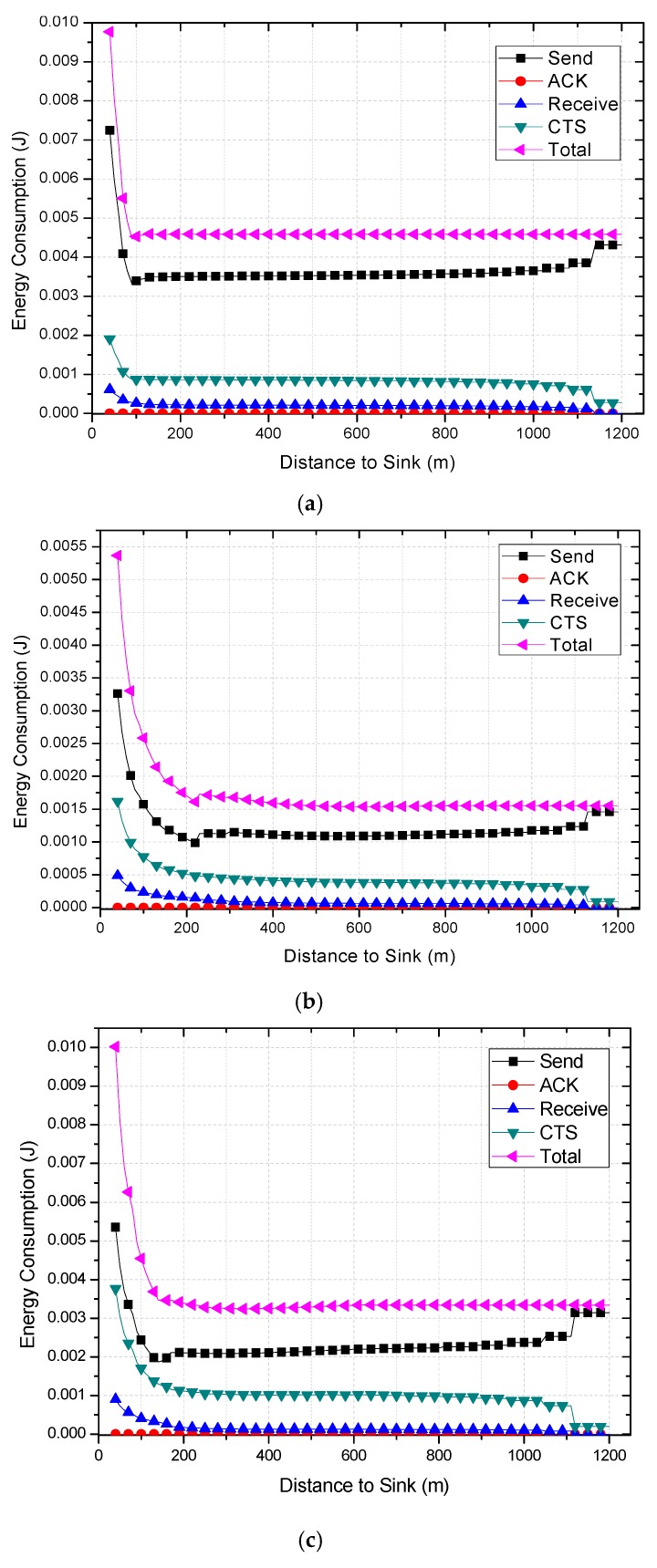

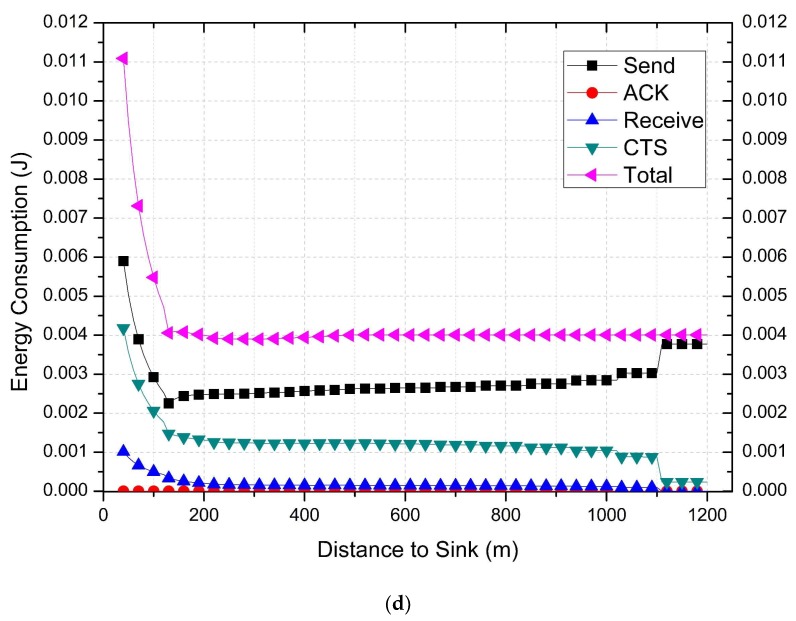
(**a**) Energy consumption of node under *r* =60, *N* = 2; (**b**) Energy consumption of node under *r* = 70, *N* = 3; (**c**) Energy consumption of node under *r* = 80, *N* = 4; (**d**) Energy consumption of node under *r* = 90, *N* = 4.

The results of energy consumptions after applying the COOR scheme are shown in this section. As can be seen in Figure 21, the energy consumption of nodes shows a rapid decline and remains stable. Additionally, the higher the previous network reliability, the smaller the drop zone.

Comparing Figure 21a with Figure 6, it is obvious that the COOR strategy increases the energy consumption of the nodes in the innermost area of the network, and maintains the energy consumption of most areas unchanged. Meanwhile, the value is equal to the maximum energy consumption of the nodes in the traditional routing method.

When it comes to the unreliable networks, the COOR scheme can still maintains the energy consumption of most areas unchanged, and the value is no more than the previous maximum energy consumption. But it needs more nodes in the near-sink region to consume more energy.

Specifically, a larger amount of data (higher data collection rate) means higher energy consumption. There is not much energy left in the nodes in the innermost region. After the reliability of the far-sink area node is greatly improved, it definitely needs to consume more energy to forward data. This causes the energy consumption of the near-sink node to be greater than the previous maximum energy consumption. In other words, with the improvement of network performance, the increase of energy consumption of nodes in near-sink region is unavoidable.

**Figure 22:**
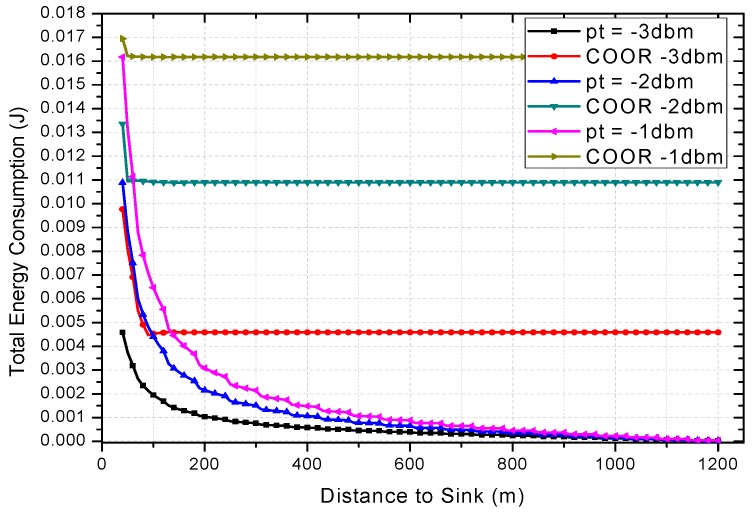
The effect of different initial transmission powers on energy consumption.

Under the default network parameters such as *r* = 60, *N* = 2, etc., the influence of the initial transmission power on the energy consumption is shown in Figure 22. From this figure, the impact of previous network reliability on the improvement of COOR scheme can be seen. When the network reliability is relatively low, the COOR scheme needs a larger energy boosting area, and a larger energy consumption increase occurs. However, when the traditional routing maintains a certain reliability, the COOR scheme will be able to make full use of the remaining energy, and only a small part of the node’s energy consumption requires a small increase. As mentioned before, although network life may be reduced, this is the inevitable price of network performance improvement.

### 6.5. Performance Comparison of COOR(R) and COOR(P) Strategy

Based on the above experimental results, both the COOR(R) strategy and COOR(P) strategy achieve great improvements to the base approach. Meanwhile, it can be observed that the former method provides a more reliable communication while the latter one focuses more on achieving low latency. Therefore, in practical applications, the COOR(R) strategy is more favorable in loss sensitive networks, especially when the reliability is the primary requirement and a certain delay can be tolerated. On the other hand, when the hypothetical scenario is delay sensitive or there is a strong need for real-time data, the COOR(P) strategy is more sensible.

For a more intuitive presentation of the different focus of the two approaches, some typical and practical examples are given in the Table 3.

## 7. Conclusions and Future Work

Guaranteeing the reliability of communication links and reducing the delay of data transmission is crucial to the application of wireless sensor networks. Opportunistic routing guarantees the reliability of data transmission at the expense of a small delay. The main innovation of this paper is based on the traditional opportunistic routing, and proposes a cross-layer optimization routing method, which improves the performance of the network significantly. The COOR scheme proposed in this paper makes full use of the residual energy of nodes to increase the transmission power, which can effectively improve the reliability of data transmission and reduce the delay. In addition, this scheme includes two types of methods, the COOR(R) method and COOR(P) method. In the COOR(R) method, after the transmission power is increased, the transmission distance is maintained so as to improve the one-hop reliability of transmissions, while the COOR(P) method increases the transmission distance with the one-hop reliability remaining unchanged. After theoretical analysis and experimental results, it is shown that the COOR scheme can significantly improve the reliability of transmissions by 36.62%, with a delay decrease rate of 21.09%.

The work of this paper aims at the network model where nodes are continuously in working state. Actually, in order to save energy, many wireless sensor networks use a duty cycle-based mode, in which nodes are periodically rotated between awake and sleep states. At the same time, nodes consume relatively less energy in sleep state, so the network life can be effectively improved in this way. However, the disadvantage of this approach is that it will lead to an increase in delay. We will further study the delay optimization of duty cycle-based WSNs in the future.

## Figures and Tables

**Table 1:** Network parameters.

Symbol	Description	Value
$R_{D}$	Sending rate/Receiving rate	19.2 kbps
$B_{N}$	Noise bandwidth	30 kHz
$P_{n}$	Background noise	−115 dBm
$d_{0}$	Reference distance	1 m
n	path loss exponent	2
$L^{s}$	Length of data packet	128 Byte
$L^{ACK}$	Length of ACK packet	8 Byte
$L^{CTS}$	Length of CTS packet	8 Byte
N	Size of candidate set	2
*R*	Network radius	1200 m
r	Transmission radius	60 m

**Table 2:** Main notations and values adopted in simulation.

Symbol	Description	Value
*R*	Network radius	1200 m
*r*	Transmission radius	60 m
$P_{t}$	Transmission power	−3 dBm
$P_{r}$	Reception power	Calculation
*N*	Number of candidate nodes	2
$R_{D}$	Sending rate/Receiving rate	19.2 kbps
$B_{N}$	Noise bandwidth	30 kHz
$P_{n}$	Background noise	−115 dBm
$d_{0}$	Reference distance	1 m
n	path loss exponent	2
$L^{s}$	Length of data packet	128 Byte
$L^{ACK}$	Length of ACK packet	8 Byte
$L^{CTS}$	Length of CTS packet	8 Byte

**Table 3:** Practical applications and method recommendation.

Application	Characteristics and Requirements	Appropriate Choice
Natural environment monitoring	Data should be accurately recorded Long transmission cycle with high delay tolerance Sensors are often evenly distributed	COOR(R) method
Wildlife tracking records	Emphasizes accurate records No need for fast transmission Small amount of data	COOR(R) method
Border monitoring of military installations	Any intrusion requires accurate reporting Tolerate minor delays	COOR(R) method
Real-time video surveillance	Requires real-time data Obsolete data is useless	COOR(P) method
Human health monitoring	Uneven distribution of sensors Obsolete data is useless Monitoring data can be merged	COOR(P) method
